# Minimal Transport Units Govern Oxygen‐Defect Stabilization and Transport for Lightweight Solid Electrolytes

**DOI:** 10.1002/advs.77283

**Published:** 2026-09-06

**Authors:** Xiaohui Li, Yongkang Zheng, Li Yang, Xiaoge Wang, Yuan Gou, Guanqun Cai, Jie Liu, Cheng Li, Kun Lin, Qiang Li, Mingxue Tang, Junliang Sun, Xianran Xing, Xiaojun Kuang

**Affiliations:** ^1^ Future Energy Interdisciplinary Center, Key Laboratory of Solid‐State Energy Conversion and Storage of Jiangxi Education Department School of Intelligent Manufacturing and Future Energy Gannan Normal University Ganzhou Jiangxi P. R. China; ^2^ Guangxi Key Laboratory of Electrochemical and Magnetochemical Functional Materials College of Chemistry and Bioengineering Guilin University of Technology Guilin P. R. China; ^3^ College of Chemistry and Molecular Engineering Beijing National Laboratory for Molecular Science (BNLMS) Peking University Beijing P. R. China; ^4^ Center for High Pressure Science and Technology Advanced Research Beijing P. R. China; ^5^ Oak Ridge National Laboratory Neutron Sciences Directorate Oak Ridge Tennessee United States of America; ^6^ Beijing Advanced Innovation Center for Materials Genome Engineering Institute of Solid State Chemistry University of Science and Technology Beijing Beijing P. R. China; ^7^ School of Materials Science and Engineering University of Science and Technology Beijing Beijing P. R. China

**Keywords:** borate oxide‐ion electrolyte, local structure, mininal transport units, NMR spectroscopy and variable‐temperature PDF, oxide‐ion transport mechanism

## Abstract

Solid electrolytes are central to electrochemical energy technologies, including fuel cells, sensors, catalysis, membrane separation, and electrolyser. However, most established oxide‐ion solid electrolytes are built around heavy B‐site cations embedded in rigid and highly connected coordination frameworks, leading to widespread high‐weight and sluggish ionic transport that is strongly coupled to large‐amplitude lattice relaxations. This intrinsic challenge hinders further performance optimization and constrains the rational design of lightweight electrolytes. Herein, we propose a minimal transport unit‐based design paradigm that combines simplified structural motifs with light‐element chemistry, enabled by the exceptional flexibility of B‐O polyhedra in coordination, rotation, deformation, and connectivity. As a proof of concept, Sc_1‐_ *_x_* Zn*_x_* BO_3‐_ *_x_*_/2_, constructed from isolated BO_3_ units, exhibits high oxide ion conductivity (σ(1000°C) ∼ 1.5 × 10^−2^ S/cm), alongside excellent thermo‐mechanical stability. Oxygen vacancies are stabilized through the formation of B_2_O_5_ units rather than isolated BO_2_ species. Long‐range oxide‐ion migration is mediated by dynamic oxygen exchange between minimal BO_3_ and B_2_O_5_ units via continuous breaking and reforming of B_2_O_5_ units, with transient BO_2_ configurations as intermediates. This study demonstrates minimal transport units as a governing principle for defect stabilization and ionic conduction in lightweight solid electrolytes, offering a general design framework for portable and scalable high‐temperature energy technologies.

## Graphical abstract

Lightweight borate chemistry combined with minimal transport units provides a new pathway for designing lightweight oxide‐ion solid electrolytes.

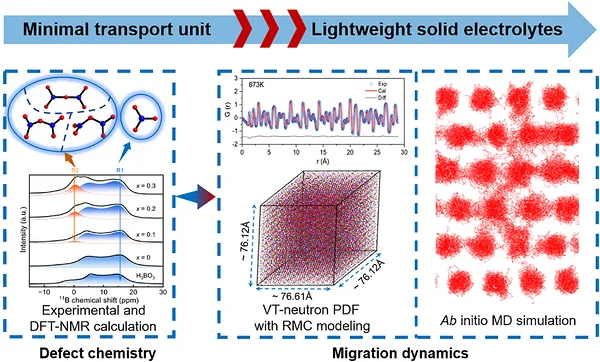

## 1 Introduction

Solid electrolytes are indispensable components in high‐temperature electrochemical energy technologies, underpinning energy conversion devices [1, 2, 3], catalysis [4, 5], membrane separation [6], high‐temperature sensing [7], and electrolyser [8]. As these technologies continue to evolve toward portable, mobile, and distributed deployment [2, 9], there is a growing demand for lightweight solid electrolytes. Oxide‐ion solid electrolytes, which exclusively transport oxide‐ions, constitute the core functional components of such devices and critically govern their efficiency, durability, and operating temperature. However, most established oxide‐ion solid electrolytes are built around heavy B‐site cations embedded in rigid and highly connected coordination frameworks [10], resulting in intrinsically widespread high‐weight and sluggish oxide‐ion migration that is strongly coupled to large‐amplitude lattice relaxations.

Over the past decades, a wide range of structural families have been developed for oxide‐ion solid electrolytes, including fluorite‐ [11], perovskite‐ [12], apatite‐ [13], scheelite‐ [14], melilite‐ [15], langasite‐ [16], and zeolite‐like [17] frameworks. Despite their structural diversity, a common characteristic across these materials is the dominance of heavy B‐site cations, such as Zr/Bi‐ [18, 19], Ga/Ge‐ [20, 21], and rare‐earth elements [22], which intrinsically increase material density and mass. Beyond this intrinsic weight constraint, an equally critical but often underappreciated limitation arises from the complexity of the ion‐transport mechanisms themselves, as these frameworks are typically constructed from highly connected polyhedral building blocks interconnected through extended three‐dimensional networks.

Early classical transport models, exemplified by the random‐walk model, treat vacancy‐ or interstitial‐mediated ion hopping as independent and uncorrelated migration behaviors [23]. Subsequent models, including the jump‐relaxation model [24] and concerted “knock‐off” mechanisms [25], incorporate interactions among mobile ions and between mobile and immobile species, allowing for correlated or simultaneous hopping processes. However, such idealized models often fail to capture the transport physics of real oxide‐ion solid electrolytes, where long‐range migration is rarely governed by simple local hopping. Instead, oxide‐ion transport is strongly coupled to collective structural distortions involving multiple polyhedral units, extended lattice relaxations, and correlated defect dynamics [26], Consequently, such complex migration mechanisms intrinsically couple oxide‐ion motion to large‐scale framework flexibility, such as rotations, deformations, and even breaking and re‐forming of anion polyhedra, resulting in high activation barriers and sluggish transport kinetics. This behavior is widely observed in highly connected frameworks such as langasite oxides [16, 27, 28] zeolite‐like structures [17], and borate‐based electrolytes [29], where effective ionic transport relies on cooperative polyhedral rearrangements or the opening and extension of tetrahedral rings.

Importantly, a unifying trend has emerged across these systems that reduced polyhedral connectivity and simplified structural building units consistently promote more efficient ionic transport. This principle is well demonstrated in layered or low‐dimensional frameworks and in structures constructed from isolated or weakly connected tetrahedral units, where ionic migration is facilitated by localized oxygen rearrangements requiring minimal lattice relaxation [14, 15, 30, 31, 32, 33]. Representative examples include layered melilite‐type oxide‐ion electrolytes (e.g. La_1.54_Sr_0.46_Ga_3_O_7.27_, σ(600°C) ∼ 0.02 S/cm) [15, 30], triple fluorite‐like layered oxyhalides (e.g. Bi_1.9_Te_0.1_LuO_4.05_Cl, σ(431°C) ∼ 0.01 S/cm) [31], apatite‐type solid electrolytes (e.g. La_9.5_(Ge_5.5_Al_0.5_O_24_)O_2_, σ(800°C) ∼ 0.16 S/cm) [32], and scheelite‐type conductors (donor‐doped Pb_1−_ *_x_* La*_x_* WO_4+_ *_x_*_/2_ (σ(800°C) ∼ 0.048 S/cm) [33]. In these frameworks, oxide‐ion transport is directly mediated by cooperative rotations and distortions of a limited number of neighboring, weakly connected polyhedral units, highlighting that simplifying the nature and connectivity of migration‐relevant structural units is a key but underexplored strategy for enhancing oxide‐ion mobility.

Inspired by this insight, we propose that combining lightweight elemental chemistry with minimal transport units, offers a powerful and underexplored design paradigm for lightweight solid electrolytes. Importantly, the minimal‐transport‐unit concept is not limited to ternary or multicomponent oxides, but is instead defined by the low coordination number, small structural unit size, and dynamic flexibility of the local motif involved in defect accommodation and ion migration. Within this framework, the borate family emerges as a particularly promising platform. Built from the lightweight element boron, borates uniquely combine low atomic mass with variable boron coordination environments and exceptional rotational and deformational flexibility of B‐O polyhedra in rotation, deformation, and connectivity [10, 29, 34, 35, 36]. These features are intrinsically favorable for accommodating oxygen defects and facilitating efficient oxide‐ion migration while minimizing structural weight.

Herein, we report a new lightweight oxide‐ion solid electrolyte, Sc_1‐_ *_x_* Zn*_x_* BO_3‐_ *_x_*_/2_, featuring isolated planar BO_3_ units, the minimal transport unit among all the reported oxide‐ion solid electrolytes. The average and local structures, as well as the associated oxide ion stabilization and migration mechanisms in Sc_1‐_ *_x_* Zn*_x_* BO_3‐_ *_x_*_/2_, were systematically studied using multiple complementary techniques, including neutron powder diffraction (NPD), Integrated Differential Phase Contrast Scanning Transmission Electron Microscopy (iDPC‐STEM), Fourier‐transform infrared (FTIR) spectroscopy, O K‐edge X‐ray absorption spectroscopy (XAS), ^11^B solid‐state nuclear magnetic resonance (NMR) spectroscopy supported by density functional theory (DFT)based NMR calculations, variable‐temperature neutron pair‐distribution function (nPDF) analysis with reverse Monte Carlo (RMC) simulations, and ab initio molecular dynamics (AIMD) simulations. We demonstrate that the oxygen vacancies were stabilized by the formation of B_2_O_5_ units rather than isolated BO_2_ species, and that long‐range migration is mediated by dynamic oxygen exchange between BO_3_ and B_2_O_5_ units.

By decoupling efficient ion transport from rigid, highly connected frameworks, this work demonstrates minimal transport units as a governing principle for defect stabilization and ionic transport in lightweight solid electrolytes. More broadly, this study provides atomic‐level insight into defect stabilization and migration, and opens a general route toward the rational design of lightweight electrolytes for next‐generation portable high‐temperature energy technologies.

## 2 Materials and Methods

Polycrystalline samples were synthesized by the high temperature solid state reaction method, with the Sc_0.7_Zn_0.3_^11^BO_2.85_ composition prepared using ^11^B isotope‐rich raw material of H_3_^11^BO_3_ (99.99%, Aladdin) for neutron powder diffraction (NPD) and neutron pair distribution function (nPDF) measurements. Details of the materials and methods, experimental procedures and data, and theoretical studies, including compound syntheses and characterizations, structural analysis, electrochemical and NMR studies, and theoretical and computational details and results are described in the SI Appendix.

## 3 Results and Discussion

### 3.1 Minimal Structural Unit Design of Sc_1‐_ *_x_* Zn*_x_* BO_3‐_ *_x_*_/2_ Electrolytes

**FIGURE 1:**
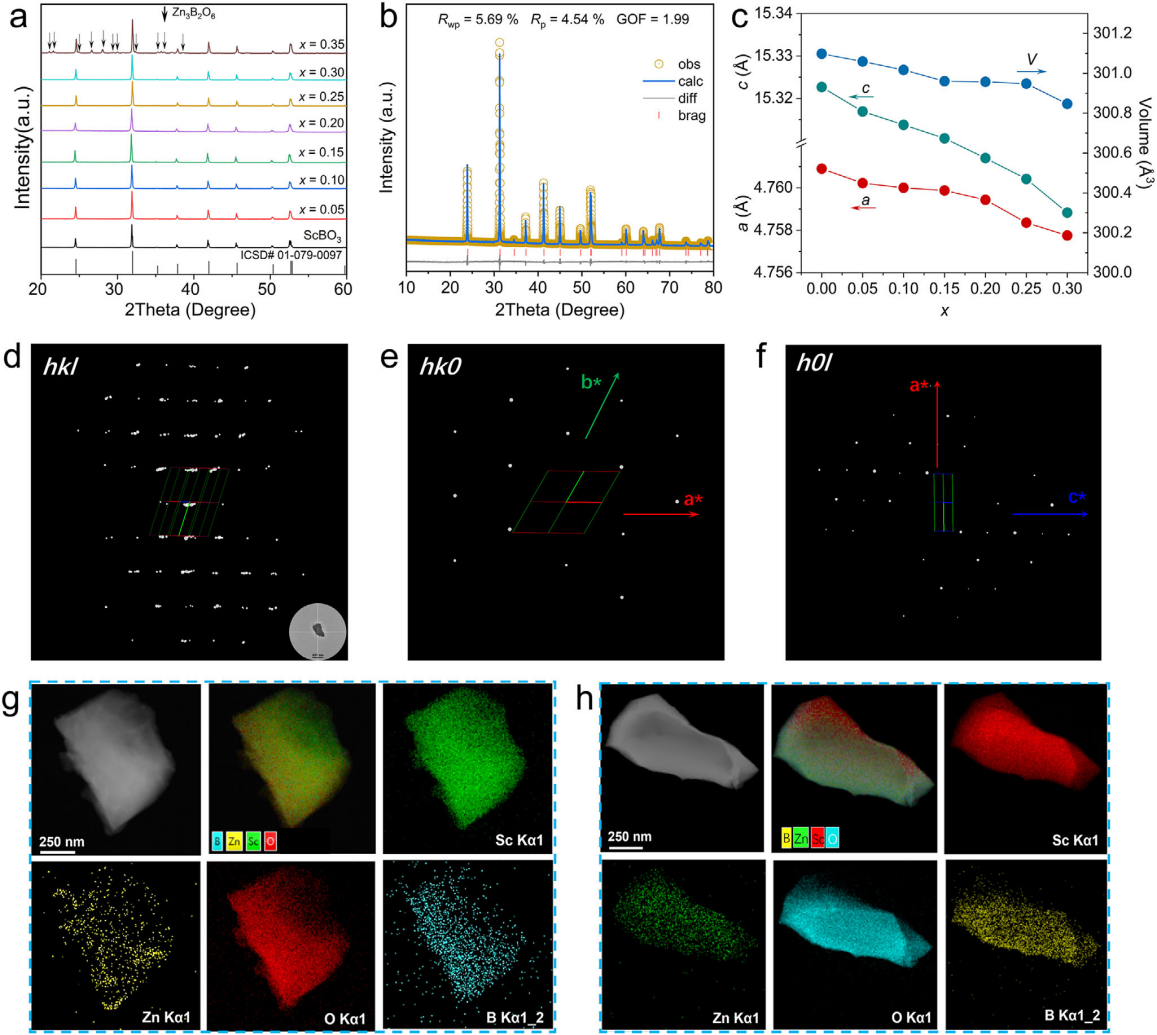
Phase formation of Sc_1‐_ *_x_* Zn*_x_* BO_3‐_ *_x_*_/2_ electrolytes. (a) XRD patterns for the 0 ≤ *x* ≤ 0.35 compositions. (b) Rietveld refinement plot of XRD data for Sc_0.7_Zn_0.3_BO_2.85_. (c) Refined cell parameters versus Zn doping concentration of *x*. (d) 3D ED reciprocal lattice of Sc_0.7_Zn_0.3_BO_2.85_ (the inset is the crystal used for data collection) and (e) (*hk*0) and (f) (*h*0*l*) slices cut from the reconstructed reciprocal lattice. (g,h) TEM elemental mapping of (g) Sc_0.85_Zn_0.15_BO_2.925_ and (h) Sc_0.7_Zn_0.3_BO_2.85_.

Sc_1‐_ *_x_* Zn*_x_* BO_3‐_ *_x_*_/2_ electrolytes are designed around isolated BO_3_ units as minimal structural building blocks to enable flexible and efficient oxide‐ion transport. Figure 1a shows the powder X‐ray diffraction (XRD) patterns of Sc_1‐_ *_x_* Zn*_x_* BO_3‐_ *_x_*_/2_ (0 ≤ *x* ≤ 0.35), demonstrating the formation of a single phase up to *x* = 0.3. When *x* > 0.3, and the appearance of the Zn_3_B_2_O_6_ impurity phase indicates that the solid solution limit in Sc_1‐_ *_x_* Zn*_x_* BO_3‐_ *_x_*_/2_ is within the range of 0 ≤ *x* ≤ 0.3. Rietveld analysis of XRD data for Sc_0.7_Zn_0.3_BO_2.85_ (Figure 1b), based on the previously reported structure of ScBO_3_ in *R* $\bar{3}$ *c* [37], converged with *R*_wp_ = 5.69%, *R*_p_ = 4.54%, GOF = 1.99, and the refined structural parameters are given in Table S2. The refined cell parameters (Figure 1c) decrease with increasing *x*, which is consistent with the substitution of smaller Zn^2+^ (r = 0.74 Å) for larger Sc^3+^ (r = 0.81 Å). Figure 1d–f shows the three‐dimensional (3D) reciprocal lattice reconstructed from the 3D electron diffraction (ED) data of Sc_0.7_Zn_0.3_BO_2.85_, which confirms the trigonal unit cell (space group: $R\bar{3}c$, *a* ∼ 4.71Å, *b* ∼ 4.71 Å, *c* ∼ 17.10 Å) with the reflection conditions *hk0*: *h* = 3n, *h* – *k* = 3n; *h0l*: *l* = 6n [37].

Transmission electron microscopy (TEM) elemental mapping data for the multiple compositions of Sc_1‐_ *_x_* Zn*_x_* BO_3‐_ *_x_*_/2_, including the parent ScBO_3_ (Figure S1), Sc_0.85_Zn_0.15_BO_2.925_ (Figures 1g and S2) and Sc_0.7_Zn_0.3_BO_2.85_ (Figures 1h, S3, and S4), confirm that the Zn^2+^ was successfully introduced into the crystal of ScBO_3_ in a homogeneous manner and exclude the possibility of the easy formation of Zn‐containing borate glass phases within Sc_1‐_ *_x_* Zn*_x_* BO_3‐_ *_x_*_/2_. Inductively coupled plasma‐atomic emission spectrometry (ICP‐OES) measurement of Sc_0.7_Zn_0.3_BO_2.85_ gives a Sc/Zn ratio close to the nominal composition, supporting the substitutional Zn^2+^ incorporation model and indicating that interstitial Zn is unlikely to be the dominant charge‐compensation mechanism. Sc^3+^ stabilizes the borate framework, whereas aliovalent Zn^2+^ substitution generates oxygen vacancies and modifies the local defect environment. Doping in ScBO_3_ and other lighter analogous with the lighter dopants may provide a similar defect‐engineering route. However, their effects are expected to depend not only on atomic mass, but also on valence, ionic radius, coordination preference, site solubility, and their influence on the rigidity and flexibility of the borate framework. Specifically, each Zn^2+^ substituting for Sc^3+^ introduces one effective negative charge ($Zn_{Sc}^{\prime }$), which is compensated by the formation of oxygen vacancies ($V_{O}^{\cdot \cdot }$). Therefore, two Zn^2+^ substitutions generate one oxygen vacancy, giving rise to the oxygen‐vacancy composition Sc_1‐_ *_x_* Zn*_x_* BO_3‐_ *_x_*_/2_. This charge‐compensation process can be described by the defect reaction equation of $2\mathrm{ZnO}+2{\mathrm{Sc}}_{Sc}^{\times }+O_{O}^{\times }\to 2Zn_{Sc}^{\prime }+V_{o}^{\cdot \cdot }+Sc_{2}O_{3}$.

### 3.2 Electrical Properties of Sc_1‐_ *_x_* Zn*_x_* BO_3‐_ *_x_*_/2_ Electrolytes

**FIGURE 2:**
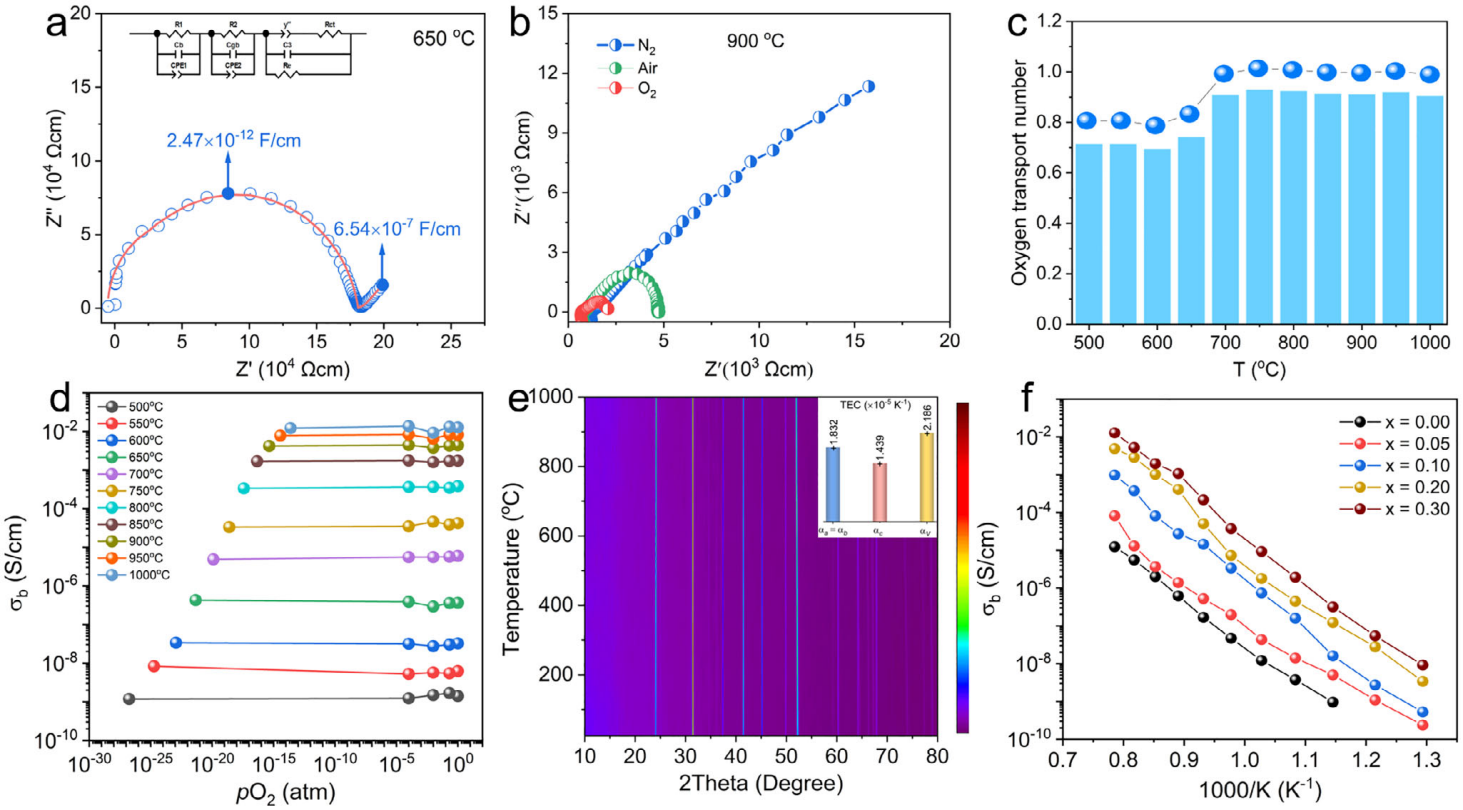
Electrical properties of Sc_1‐_ *_x_* Zn*_x_* BO_3‐_ *_x_*_/2_ electrolytes. (a) Typical complex impedance plot of Sc_0.7_Zn_0.3_BO_2.85_ at 650°C. (b) Complex impedance plots of Sc_0.7_Zn_0.3_BO_2.85_ at 900°C under different oxygen partial pressures (pure N_2_, air, and pure O_2_). (c) Oxygen transport number of Sc_0.7_Zn_0.3_BO_2.85_. (d) Bulk conductivities of Sc_0.7_Zn_0.3_BO_2.85_ measured under different oxygen partial pressures. (e) Variable‐temperature XRD patterns of Sc_0.7_Zn_0.3_BO_2.85_, with the inset showing the thermal expansion coefficients of Sc_0.7_Zn_0.3_BO_2.85_ derived from VT‐XRD data. (f) Arrhenius plots of bulk conductivities in air as a function of Zn doping concentration.

To study oxygen vacancy migration in the structural framework of trigonal ScBO_3_, which features the minimal BO_3_ planar unit, alternating current (AC) impedance spectroscopy measurements were conducted on Sc_1‐_ *_x_* Zn*_x_* BO_3‐_ *_x_*_/2_ ceramics in air. In contrast to the parent ScBO_3_, whose complex impedance plots display only bulk and grain boundary components (*C*_b_ (900°C) = 2.87 × 10^−12^ F/cm, Figure S5), the complex impedance plot of Sc_0.7_Zn_0.3_BO_2.85_ at 650°C (Figure 2a) shows an apparent Warburg‐type electrode response associated with ionic conduction, alongside a semielliptical arc [38]. The data were modeled using an equivalent circuit comprising the contributions of the bulk and grain‐boundary responses with the Warburg electrode response, where the parallel *R*//*C*//*CPE* ^*^ circuits in series were used for analyzing the non‐ideally semicircular arcs for bulk and grain‐boundary components [39, 40, 41]. With increasing temperature, the electrode response gradually collapsed down to semicircular arc (Figure 2b).

To further identify the charge carrier species for the ionic conduction in Sc_0.7_Zn_0.3_BO_2.85_, the AC impedance measurements on the Sc_0.7_Zn_0.3_BO_2.85_ ceramic at different oxygen partial pressures were performed at 900°C (Figure 2b), which showed that the electrode response arc collapsed to semicircular arcs in pure O_2_ gas, air, and reverted to a Warburg‐type response in pure N_2_ gas. This oxygen partial pressure sensitivity of electrode response [42] suggests that oxide ions are the primary charge carriers. At low oxygen partial pressure (e.g., pure N_2_, Figure 2b), limited oxide ion diffusion coefficients (1/2O_2_ + 2e ↔ O^2−^) and high charge transfer resistance result in an inclined line‐shaped Warburg‐type response. In contrast, higher oxygen partial pressures (e.g., air, pure O_2_, Figure 2b) enhance oxide ion diffusion coefficients and reduce charge transfer resistance, leading to the collapse of the response into small semicircular arcs. Electromotive force (EMF) measurements using an oxygen concentration cell method were employed to determine the oxygen transport numbers ($t_{O^{2-}}$) of the parent ScBO_3_ and Sc_0.7_Zn_0.3_BO_2.85_. In contrast to the near‐zero oxygen transport number of the parent ScBO_3_ (Figure S6), the composition Sc_0.7_Zn_0.3_BO_2.85_ ceramic maintained an oxygen transport number of approximately 90% over the temperature range of 700°C–1000°C (Figure 2c), further demonstrating that Sc_0.7_Zn_0.3_BO_2.85_ is a pure oxide‐ion solid electrolyte with negligible electronic conduction.

The bulk conductivities of Sc_0.7_Zn_0.3_BO_2.85_ remain stable over a wide oxygen partial pressure range of 1–10^−27^ atm (Figure 2d), further confirming the pure ionic conduction. Variable temperature X‐ray diffraction (VT‐XRD) measurement (Figures 2e and S7) demonstrates excellent thermal stability for Sc_0.7_Zn_0.3_BO_2.85_ over the temperature range of 25°C–1000°C. The thermal expansion coefficients (TEC) of Sc_0.7_Zn_0.3_BO_2.85_, derived from the VT‐XRD patterns (inset of Figure 2e), are as follows: *α*_a_ = *α*_b_ ≈ 1.832 × 10^−5^ K^−1^, *α*_c_ ≈ 1.439 ×10^−5^ K^−1^, and *α*_v_ ≈ 2.186 × 10^−5^ K^−1^. Notably, the volume thermal expansion coefficient of Sc_0.7_Zn_0.3_BO_2.85_ shows a weak thermal expansion with a level of *α*_v_ ≈ 2.186 × 10^−5^ K^−1^, which matches well with the dominant electrode materials in the operating temperature range, such as the cobalt‐containing perovskites [43, 44, 45, 46] (Sm_0.5_Sr_0.5_CoO_3‐_ *_x_*, (La,Sr)(Co,Fe)O_3‐_ *_x_*, Ba_0.5_Sr_0.5_Co_0.8_Fe_0.2_O_3‐_ *_x_* (BSCF) and SrNb_0.1_Co_0.9_O_3‐_ *_x_* (SNC), with TEC values of *α*_v_ ≈ 2–2.5 × 10^−5^ K^−1^). The conductivities of Sc_0.7_Zn_0.3_BO_2.85_ (Figure 2f) at 1000°C reach ∼1.5 × 10^−2^ S/cm, showing an enhancement in oxide ion conductivity of about three orders compared to the parent ScBO_3_ (σ(1000°C) ∼ 1.2 × 10^−5^ S/cm). It is interesting to note here that the pristine and Zn‐doped samples exhibit broadly comparable activation energies from the Arrhenius plots. However, the similar slopes do not necessarily indicate identical conduction behavior. For pristine ScBO_3_, no measurable oxide‐ion conduction response is observed within the experimental detection limit. Therefore, the conduction in pristine ScBO_3_ is predominated by electronic conduction, and its activation energy is 1.89 eV, roughly half of the bandgap (4.148 eV from DFT calculation in Figure S9; 4.0 eV from UV–vis absorption measurement [47]) for ScBO_3_. This indicated that the electronic conduction in the pristine ScBO_3_ is mainly from the transition over the intrinsic bandgap. In contrast, Zn^2+^ substitution for Sc^3+^ introduces oxygen vacancies, thereby activating oxide‐ion transport, which predominates the conduction in the doped borate framework. Therefore, the observation on activation energy for ionic conduction in the doped ScBO_3_ is comparable to that for the electronic conduction in the pristine ScBO_3_ is a coincidence.

Furthermore, the oxide ion conductivities of Sc_0.7_Zn_0.3_BO_2.85_ are approximately 2 orders of magnitude higher than those of (Y/Gd)_1‐_ *_x_* Zn*_x_* BO_3‐_ *_x_*_/2_ containing B_3_O_9_ tetrahedral rings (e.g. σ(900°C) ∼ 8 × 10^−4^ S/cm) [29], demonstrating that the “simplify structural unit” strategy in borates facilitates and enhances oxide ion transport. Although the conductivity remains lower than that of state‐of‐the‐art oxide‐ion electrolytes, such as doped fluorite and LaGaO_3_‐based systems [10], it represents a significant improvement among borate‐based oxide‐ion conductors and highlights the potential of minimal transport units as an alternative strategy for designing lightweight oxide‐ion electrolytes.

### 3.3 Average and Defect Structures of Sc_1‐_ *_x_* Zn*_x_* BO_3‐_ *_x_*_/2_ Electrolytes

**FIGURE 3:**
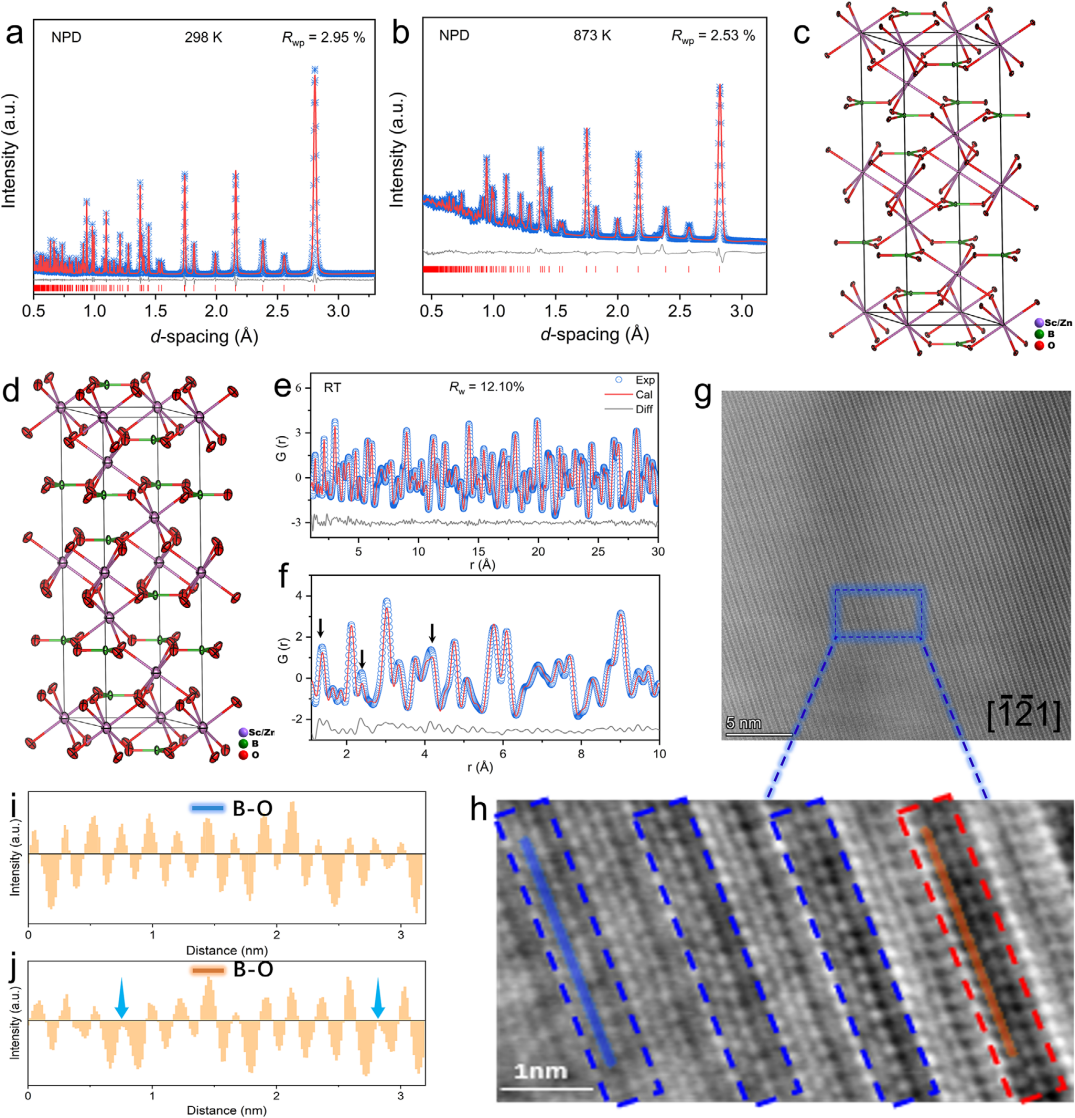
Structure of Sc_0.7_Zn_0.3_BO_2.85_ electrolyte. (a, b) Rietveld refinement plots of NPD data at 298 and 873 K, respectively. (c, d) Ellipsoid plots of the framework atoms viewed along the *b*‐axis at 298 and 873 K, respectively. (e) Small‐box nPDF fitting with (f) the short‐range fitting in the range of 1–10 Å, where the black arrows in (f) indicate mismatches. (g) iDPC‐STEM image viewed along the [$\bar{1}\bar{2}1$] direction. (h) High‐magnification iDPC‐STEM image for the region marked in (g). (i, j) Intensity profile analysis along the blue and red solid lines as marked in (h), respectively.

As neutron diffraction is more sensitive to the light B and O atoms than x‐ray diffraction, and the natural B has strong absorption to the neutrons given the isotope ^10^B absorbs neutrons severely, the much less neutron‐absorption isotope ^11^B‐rich Sc_0.7_Zn_0.3_^11^BO_2.85_ samples were prepared for the variable‐temperature NPD and nPDF data collections at room temperature (RT = 298 K) and high temperature (HT = 873 K) for analysis of average and local structures. Rietveld analysis of the NPD data for Sc_0.7_Zn_0.3_^11^BO_2.85_ at room temperature (Figure 3a and Table 1) indicates that all the atomic sites are fully occupied except for the oxygen site (*occ* = 0.918(3), Table 1), and the refined composition Sc_0.718(2)_Zn_0.282(2)_^11^BO_2.753(3)_ is consistent with the nominal composition. The Rietveld refinements of NPD data confirm the existence of oxygen vacancies and did not show apparent evidence of extra Zn as interstitial defects for charge compensation to Zn^2+^ substitution for Sc^3+^. The refined anisotropic atomic displacement parameters (ADPs) and the derived ellipsoid plots of the framework atoms viewed along the *b*‐axis at 298 K are given in Table S3 and Figure 3c, respectively.

**TABLE 1:** The final refined structural parameters of Sc_0.7_Zn_0.3_BO_2.85_ from NPD data at 298 K^*^.

Atom	Site	*x*	*y*	*z*	occ.	U_iso_ (Å^2^)
Sc1	6*b*	0	0	0	0.718 (2)	0.0027(1)
Zn1	6*b*	0	0	0	0.282 (2)	0.0027(1)
B1	6*a*	0	0	0.25	1	0.0025(2)
O1	18*e*	0.28958(9)	0	0.25	0.918(3)	0.0040(1)

^*^Space group: *R* $\bar{3}$ *C*, *a* = *b* = 4.75745(2) Å, *c* = 15.3214 (1) Å, *α* = *β* = 90°*, γ* = 120°, *V* = 300.316 (3) Å^3^.

At high temperature, the structure of Sc_0.7_Zn_0.3_^11^BO_2.85_ shows similar average structural parameters, as determined by the Rietveld analysis (Figure 3b and Table S4). However, the refined ADPs at high temperature (Figure 3d and Table S5) reveal more positional disorder, with significant anisotropic elongation of the oxygen ellipsoids along the *ab* plane at oblique and randomly varying angles, indicating 3D migration potential for oxide ions in Sc_0.7_Zn_0.3_BO_2.85_. The small‐box refinement of the total scattering neutron PDF data of Sc_0.7_Zn_0.3_^11^BO_2.85_ at room temperature (Figure 3e) gave the same average structure, but with short‐range mismatches as indicated by black arrows (Figure 3f). These mismatches presumably arise from the presence of oxygen vacancies, resulting in variations in B─O bond lengths and coordination numbers in the local structure of Sc_0.7_Zn_0.3_BO_2.85_.

Integrated differential phase contrast scanning transmission electron microscopy (iDPC‐STEM) offers high atomic resolution for solid electrolytes by improving electron utilization efficiency, reducing the required electron dose and enabling high‐contrast imaging of light elements [48]. Therefore, we employed the iDPC‐STEM imaging technique to probe the oxygen defects in Sc_0.7_Zn_0.3_BO_2.85_. The iDPC‐STEM images of Sc_0.7_Zn_0.3_BO_2.85_ at low and high magnifications viewed along [$\bar{1}\bar{2}1$] direction are given in Figure 3g,h, respectively. Intensity profile analysis along the blue (Figure 3i) and orange (Figure 3j) lines labeled in Figure 3h for the iDPC images reveals the shortest distances between the spots of 1.3 and 2.5 Å, corresponding to the B─O and B─B bond lengths, respectively, which is consistent with the projected bond lengths in the structure along the [$\bar{1}\bar{2}1$] direction. In contrast, the intensity profile analysis along the orange line (Figure 3j) shows intensity absences at oxygen atom positions, as compared to the blue lines (Figure 3i). This provides direct evidence for the formation of oxygen vacancies in Sc_0.7_Zn_0.3_BO_2.85_.

**FIGURE 4:**
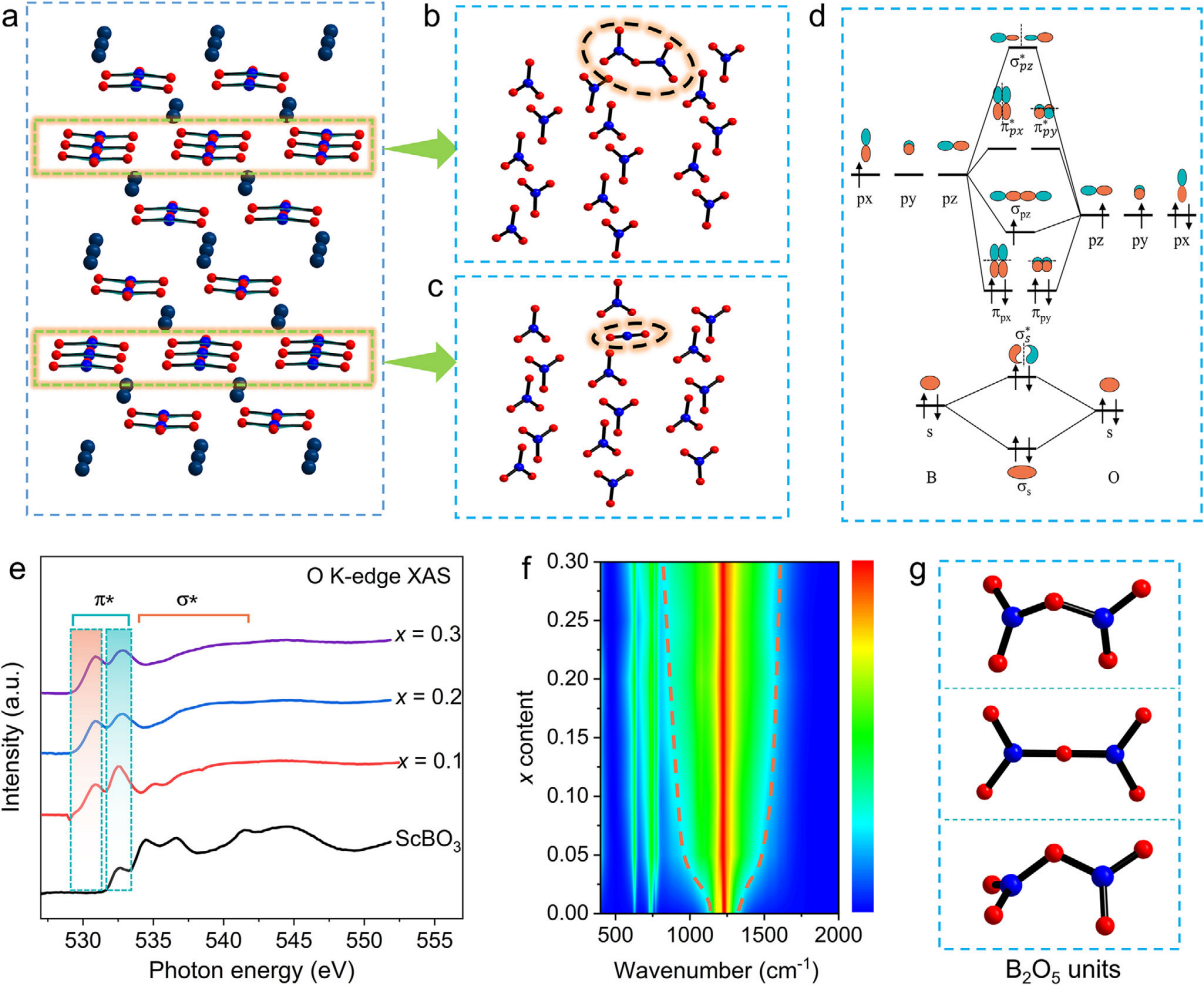
Oxygen vacancy stabilization mechanism revealed by geometry optimization and spectroscopic characterization techniques. (a) Structure of Sc_0.7_Zn_0.3_BO_2.85_ viewed along [010] direction. (b,c) Relaxed structures surrounding oxygen vacancies, highlighting (b) B_2_O_5_ and (c) BO_2_ units. (d) Molecular orbital diagram of B─O bonding interactions. (e) Ultrasoft O K‐edge XAS spectra of Sc_1‐_ *_x_* Zn*_x_* BO_3‐_ *_x_*_/2_ (0 ≤ *x* ≤ 0.3). (f) Fourier‐transform IR spectra of Sc_1‐_ *_x_* Zn*_x_* BO_3‐_ *_x_*_/2_ (0 ≤ *x* ≤ 0.3), and the broadened infrared absorption peaks marked by orange lines represent the absorption of (g) B_2_O_5_ units with various distortions.

To better understand the stabilization mechanism of oxygen vacancies in Sc_1‐_ *_x_* Zn*_x_* BO_3‐_ *_x_*_/2_, DFT‐based geometry optimizations combined with spectroscopic analyses were carried out. A 2 × 2 × 3 supercell structural model of Sc_0.7_Zn_0.3_BO_2.85_ containing oxygen vacancies was constructed for geometry optimization. The relaxed structures around the oxygen vacancies (Figure 4a–c) indicate that the oxygen vacancies could be stabilized by forming B_2_O_5_ (Figure 4b) and/or BO_2_ (Figure 4c) units.

As shown in the molecular program for B‐O (Figure 4d), the *π* ^*^ antibonding state feature is uniquely related to 3‐coordinated boron environments. The oxygen 1s core electron transitions to *π* ^*^ antibonding states can be unequally separated into contributions from bridging oxygen and non‐bridging oxygen [49]. Given the sensitivity of the oxygen K‐edge to the electronic structure associated with short‐ and extended‐range bonding, ultrasoft x‐ray absorption spectroscopy (XAS) for K‐edges of low‐Z O element is a powerful technique for probing the local environment of oxygen atoms, particularly changes in B─O bonding involving terminal and bridging oxygens. The O K‐edge XAS spectra of Sc_1‐_ *_x_* Zn*_x_* BO_3‐_ *_x_*_/2_ (0 ≤ *x* ≤ 0.3, Figure 4e), calibrated using Au as a reference (Figure S8), show the characteristic electronic transitions from the oxygen 1s core state to the narrow *π* ^*^ and broader σ^*^ antibonding states associated with the BO_3_ units. The peak observed at ∼532.6 eV corresponds to oxygen 1s core electron transitions to $\pi_{px}^{*}$ antibonding state in BO_3_ units associated with terminal oxygen, consistent with the XAS spectrum of parent ScBO_3_ (Figure 4e bottom). Whereas the new peak observed at 530.8 eV, shifted by ∼1.8 eV, corresponds to the electronic transitions toward the same antibonding $\pi_{py}^{*}$ but associated with the newly formed B_2_O_5_ units containing bridging oxygens, as also revealed by the differences between PDOS of O in the parent ScBO_3_ and Sc_0.7_Zn_0.3_BO_2.85_ (Figure S9). The downshift of the π∗ peak in the doped compositions Sc_1‐_ *_x_* Zn*_x_* BO_3‐_ *_x_*_/2_ is attributed to the weakened of B─O bonding energy resulting from the formation of the B_2_O_5_ geometry, as evidenced by the slight increase in bond length (BO_3_: ∼ 1.37 Å; B_2_O_5_ ∼ 1.39 Å) [34]. Similar spectral signatures coincide with the reported variations in lithium borates [50, 51, 52].

Complementary evidence is provided by Fourier‐transform infrared (FTIR) spectroscopy. The FTIR spectrum of parent ScBO_3_ (Figure 4f) contains only the vibrational absorption peaks typical of BO_3_ units [53]. With increasing the oxygen vacancy concentration, additional broadened infrared absorption peaks emerge within the 800–1500 cm^−1^ range, as marked by the orange lines in Figure 4f, indicating the formation of B_2_O_5_ and/or BO_2_ units in the doped compositions Sc_1‐_ *_x_* Zn*_x_* BO_3‐_ *_x_*_/2_. Although these findings provide valuable insights, it remains an open question whether the stabilization mechanism for oxygen vacancies in Sc_1‐_ *_x_* Zn*_x_* BO_3‐_ *_x_*_/2_ is predominantly governed by the formation of B_2_O_5_ or BO_2_ units.

### 3.4 Experimental and DFT‐Based NMR Calculations for Sc_1‐_ *_x_* Zn*_x_* BO_3‐_ *_x_*_/2_

**FIGURE 5:**
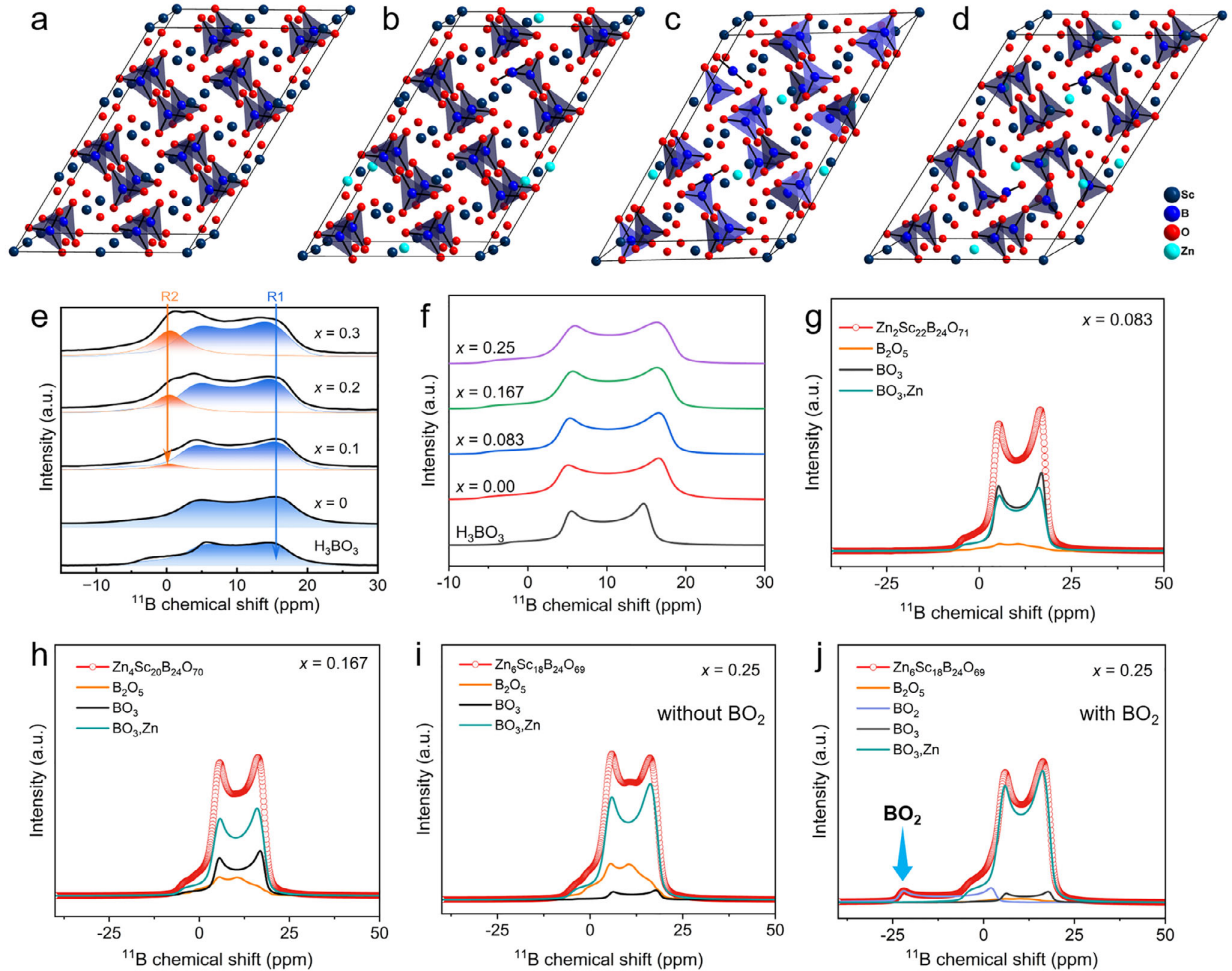
Experimental deconvolution and DFT‐based ^11^B NMR calculations for Sc_1‐_ *_x_* Zn*_x_* BO_3‐_ *_x_*_/2_. (a‐d) Typical structural models employed in the DFT calculations for Sc_1‐_ *_x_* Zn*_x_* BO_3‐_ *_x_*_/2_: (a) *x* = 0, (b) *x* = 0.083, (c) *x* = 0.167 and (d) *x* = 0.25. (e) Experimental solid‐state ^11^B NMR spectra and (f) DFT‐based NMR calculated and simulated ^11^B NMR spectra. (g–j) Simulated ^11^B NMR spectra of Sc_1‐_ *_x_* Zn*_x_* BO_3‐_ *_x_*_/2_ (*x* = 0.083, 0.167, 0.25) compositions, highlighting contributions from different structural units (g–i) without BO_2_ units and (j) with BO_2_ units, where (BO_3_, Zn) units denote BO_3_ units with different numbers of Zn atom neighbors in the second coordination sphere.

To gain deeper insight into whether the local structure responsible for oxygen vacancy stabilization in Sc_1‐_ *_x_* Zn*_x_* BO_3‐_ *_x_*_/2_ is dominated by B_2_O_5_ or BO_2_ units, solid‐state ^11^B NMR technique together with DFT‐based ^11^B NMR calculations was used to probe the oxygen vacancy‐induced variations in the local structure. The deconvolution of the experimental ^11^B‐NMR data of Sc_1‐_ *_x_* Zn*_x_* BO_3‐_ *_x_*_/2_ (Figure 5a) revealed two main common resonance signals (Table S6), R1 (∼ 21 ppm) and R2 (∼ 0.5 ppm), in the Zn‐doped compositions Sc_1‐_ *_x_* Zn*_x_* BO_3‐_ *_x_*_/2_. As Zn concentration *x* increased, the R1 signal weakened, while the R2 signal strengthened. In addition, the overall ^11^B‐NMR signal peak exhibits progressive broadening with increasing *x*, indicating increased local structural complexity. The complex features of the ^11^B NMR spectra make it very difficult to identify the ^11^B coordination environments if based on the empirical knowledge alone.

To accurately reveal the subtle variations in the local environment of B in Sc_1‐_ *_x_* Zn*_x_* BO_3‐_ *_x_*_/2_ through the ^11^B solid‐state NMR data, we performed periodic DFT‐based ^11^B NMR calculations for different compositions of Sc_1‐_ *_x_* Zn*_x_* BO_3‐_ *_x_*_/2_ (*x* = 0, 0.083, 0.167, and 0.25) to simulate the possible defect structures and calculate the chemical shielding constants and electric field gradient (EFG) tensors. Geometry optimization of structural models (Figure 5a–d) revealed that for *x* = 0.083 composition, all structural models (Table S7) have equivalent final energies and show both BO_2_ and BO_3_ coordination environments. For *x* = 0.167 and 0.25 compositions, the lower‐energy models incorporated both BO_2_ and BO_3_ units along with B_2_O_5_ units (Table S7). These results indicate that at lower oxygen vacancy concentrations, BO_2_ units were formed to stabilize oxygen vacancies, while at higher oxygen vacancy concentrations, B_2_O_5_ units become dominant.

The calculated chemical shift distributions for ^11^B nuclei in different coordination environments, including the presence of Zn atoms in their secondary coordination spheres, in Sc_1‐_ *_x_* Zn*_x_* BO_3‐_ *_x_*_/2_ (*x* = 0, 0.083, 0.167, and 0.25) are summarized in Table S8. For BO_3_ units, the average chemical shift is 21.26 ppm (Table S9), while BO_3_ units with Zn atoms in the second coordination sphere (denoted as BO_3_, Zn) shift slightly lower to 21.01 ppm (Table S9). Both BO_3_ unit and (BO_3_, Zn) units (Figure 5g–j) show comparable broadening (∼1 ppm difference) and double‐peak splitting due to quadrupolar coupling interactions, with approximately the same peak intensity. These double peaks have similar relative intensities, with left peak has lower intensity, and right peak has higher intensity. In contrast, the isotropic chemical shifts for BO_2_ and B_2_O_5_ units are 9.93 and 19.23 ppm, respectively (Table S9). The variations observed in the overall NMR spectra with increasing Zn doping are attributed to the formation of the B_2_O_5_ units and/or the BO_2_ units, as mentioned in the structural optimization results.

The simulated NMR spectrum of parent ScBO_3_, containing only BO_3_ units perfectly reproduces the experimental spectrum (Figure S10a), which validates the correctness and plausibility of the DFT‐based NMR calculations. However, when BO_2_ units were included in the simulated NMR spectra for Zn‐doped compositions (Figures 5j and S10b,c), extra characteristic low‐shift signals (∼ 25 ppm) were produced. These extra signals at ∼ 25 ppm, highlighted by a blue arrow in Figures 5f and S10b,c, were not observed in the experimental data. Conversely, the simulated ^11^B NMR spectra of Sc_1‐_ *_x_* Zn*_x_* BO_3‐_ *_x_*_/2_ (*x* = 0, 0.083, 0.167, 0.25, Figure 5f) compositions with contributions from different structural units but without BO_2_ units well reproduced the experimental ^11^B NMR data (Figure 5e), especially when compared to the simulated spectra of Sc_1‐_ *_x_* Zn*_x_* BO_3‐_ *_x_*_/2_ (*x* = 0.25) composition without BO_2_ (Figure 5i) and with BO_2_ units (Figure 5j). This result confirms that the oxygen vacancies in Sc_0.7_Zn_0.3_BO_2.85_ were stabilized by the formation of B_2_O_5_ units rather than BO_2_ units. This assignment is further supported by the relative‐energy analysis, which identifies B_2_​O_5_​‐containing configurations among the energetically favorable defect models (Table S7). Moreover, simulations based on the lowest‐energy model for each composition retain the principal spectral assignments (Tables S10–S12 and Figure S11), further supporting B_2_​O_5_​ rather than BO_2_​ as the dominant oxygen‐vacancy‐stabilizing environment.

In short, according to the above analysis from the geometry optimization and spectroscopic characterization techniques (e.g., the ultrasoft O K‐edge XAS and Fourier‐transform IR spectra, 3.3 Section), the oxygen vacancies could be stabilized by the formation of B_2_O_5_ and/or BO_2_ units. However, the combination of the experimental NMR spectra and DFT‐based NMR calculations reveal that the actual oxygen vacancy stabilization mechanism is predominantly governed by the formation of B_2_O_5_ units rather than BO_2_ units in Sc_0.7_Zn_0.3_BO_2.85_. This combination of experimental and DFT‐based NMR calculations not only gives a real oxygen vacancy stabilization mechanism but is also extremely instructive in identifying subtle variations in the spectroscopic characterization techniques, such as the XAS peak at 530.8 eV in Figure 4e is attributed to the B_2_O_5_ unit, and the additional broadened infrared absorption peaks within the 800–1500 cm^−1^ range in Figure 4f arise from the formation of B_2_O_5_ units with various distortions (Figure 4g) rather than BO_2_ units. Furthermore, this combination of experimental and DFT‐based NMR calculations bridges the gap between other related local structure characterization techniques.

### 3.5 Oxygen Vacancy Transport Mechanism in Sc_1‐_ *_x_* Zn*_x_* BO_3‐_ *_x_*_/2_ Electrolyte

**FIGURE 6:**
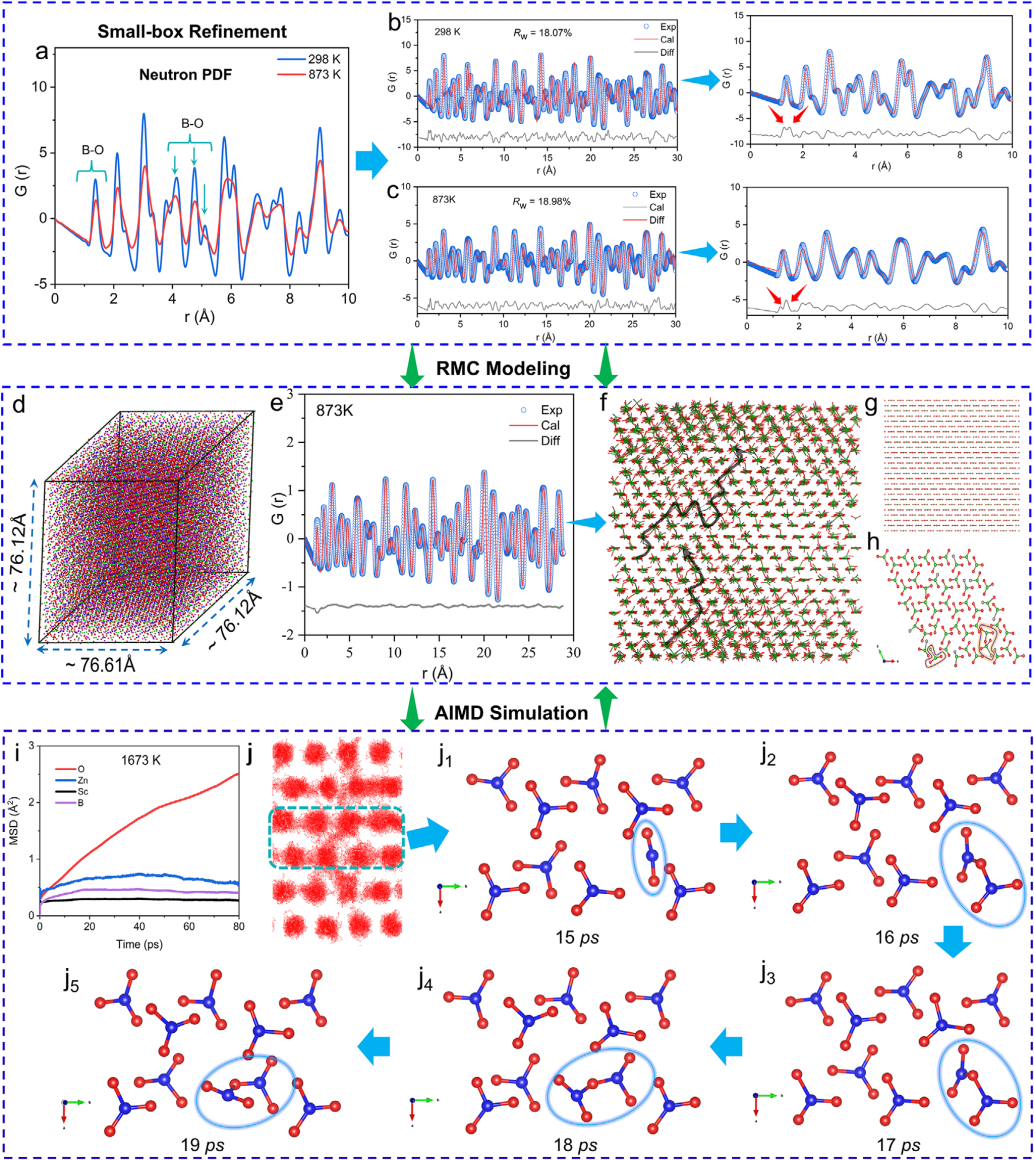
Oxygen vacancy migration mechanism in Sc_1‐_ *_x_* Zn*_x_* BO_3‐_ *_x_*_/2_ electrolytes_._ (a) Variable‐temperature nPDF data. Small‐box refinements of nPDF data at (b) 298 K and (c) 873 K. (d) 3D atomic configuration of the refined supercell, approximately 76.12Å × 76.12 Å × 76.61 Å. (e) Big‐box refinements of nPDF data at 873 K. (f, h) 3D distribution of B‐O coordination environments in the refined supercell from RMC simulation, viewed along (f) the [100] and (h) the [001] direction, where the black arrows in (f) indicate oxygen exchange pathways facilitated by displacement and deformation of B‐O polyhedra, compared to (g) the initial structural model viewed along [100] direction. (i‐j) AIMD simulations at 1673 K: (i) mean square displacements of atoms, (j) scatter plot of oxide ion positions, and (j_1‐5_) the evolution of B‐O configurations.

The atom pair distribution function (PDF) based on the neutron total scattering technique enables probing the short‐range local structures that are masked by long‐range structures. In this study, variable temperature neutron total scattering technique was used to capture the local disorder and short‐range correlations associated with the stabilization and migration of oxygen vacancies in Sc_0.7_Zn_0.3_^11^BO_2.85_ at operating temperature. Figure 6a shows the variable temperature nPDF data of Sc_0.7_Zn_0.3_^11^BO_2.85_ in the range of 0–10 Å, revealing significant peak broadening associated with B─O and Sc/Zn─O bond lengths as temperature increases from 298 to 873 K. Small‐box refinements of variable temperature nPDF data (Figure 6b,c) show poor fits and mismatches in the short‐range fitting region (marked by red arrows), indicating the occurrence of local structural disorder or distortion in Sc_0.7_Zn_0.3_^11^BO_2.85_ at high temperatures.

To explore the spatial variation of atomic distributions in Sc_0.7_Zn_0.3_^11^BO_2.85_ at high temperature, neutron PDF data at 873 K were analyzed using RMC modeling based on a 16 × 16 × 5 supercell (76.12Å × 76.12 Å × 76.61 Å, Figure 6d,e). The refined 3D distributions of B and O atoms in Sc_0.7_Zn_0.3_^11^BO_2.85_ along the [100] direction (Figure 6f) reveals substantial local displacements and distortions of B‐O polyhedra in comparison to the initial structure (Figure 6g). The black arrows in Figure 6f indicate possible oxygen‐defect migration‐related structural connections inferred from the experimentally constrained local configurations. Furthermore, along the [001] direction (Figure 6h), the formation of B_2_O_5_ and BO_2_ units at 873 K is evident. This is attributed to the rotational and deformation flexibility of isolated BO_3_ planar units, which allow the formation of structural intermediates conducive to oxygen exchange, thereby facilitating oxide ion migration in Sc_0.7_Zn_0.3_^11^BO_2.85_. Therefore, the RMC modeling based on neutron PDF data reveals the high‐temperature local structural landscape associated with oxygen‐defect evolution, while the dynamic role of these configurations in oxide‐ion migration is further investigated by AIMD simulations.

To elucidate the dynamics of oxygen‐vacancy migration in the structural framework of Sc_1‐_ *_x_* Zn*_x_* BO_3‐_ *_x_*_/2_ on a temporal scale, ab initio molecular dynamics (AIMD) simulations were performed for Sc_1‐_ *_x_* Zn*_x_* BO_3‐_ *_x_*_/2_ at 1473, 1573, and 1673 K (Figure 6i, Table S1, Figures S12 and S13, and Video File S1). The mean square displacement (MSD) values of Sc, Zn, B, and O atoms (Figures 6i and S12a–S13a) demonstrate that oxygen exhibits pronounced long‐range diffusion, whereas the cations mainly undergo thermal vibrations, confirming oxygen‐vacancy‐mediated oxide‐ion transport.

Beyond the oxygen‐exchange event initially identified within the 15–19 ps time window at 1673 K (Figure 6j and Video S1), similar oxygen exchange processes were observed in multiple independent trajectory segments at different temperatures (Figures S12–S13), indicating that the observed migration behavior represents a recurrent dynamic process rather than an isolated event. Furthermore, the calculated oxygen diffusion coefficients increase systematically with temperature, reaching 2.21 × 10^−7^, 3.23 × 10^−7^, and 4.63 × 10^−7^ cm^2^/s at 1473, 1573, and 1673 K, respectively, further supporting the thermally activated oxygen migration process.

To understand the atomic‐scale origin of this migration behavior, the oxygen‐exchange event at 1673 K within the 15–19 ps time interval was further analyzed (Figure 6j and Video S1). Specifically, a transient BO_2_‐like unit interacts with an adjacent BO_3_ unit to form a B_2_O_5_ unit (Figure 6j_1_‐_2_). This B_2_O_5_ unit subsequently exchanges oxygen with another adjacent BO_3_ unit, breaking and reforming into new B_2_O_5_ and BO_3_ units (Figure 6j_3‐5_). This continuous breaking and reformation of B_2_O_5_ and BO_3_ units drives long‐range oxide ion migration. The transient BO_2_‐like configurations observed during AIMD simulations further support the high‐temperature local structural features identified by nPDF‐RMC analysis.

The lightweight oxide‐ion solid electrolyte Sc_0.7_Zn_0.3_BO_2.85_ presented here demonstrates the feasibility of the “minimal transport unit and lightweight chemistry” design paradigm proposed in this work. In contrast to conventional electrolytes built from heavy cations and strongly connected polyhedral frameworks, this material exploits the intrinsically light and flexible borate chemistry to realize oxide‐ion transport mediated by minimal functional units. The minimal planar BO_3_ and defect‐associated B_2_O_5_ structural units provide favorable local environments for oxygen‐vacancy stabilization and migration. The convergence of diffraction, spectroscopy, solid‐state NMR, variable‐temperature neutron PDF techniques, and AIMD simulations establishes an atomic‐scale understanding of oxygen‐defect stabilization and transport in this borate framework.

## 4 Conclusion

In summary, we propose a “minimal transport unit and lightweight chemistry” design paradigm for oxide‐ion solid electrolytes and demonstrate its feasibility through the development of the lightweight solid electrolytes Sc_1‐_ *_x_* Zn*_x_* BO_3‐_ *_x_*_/2_, constructed from isolated planar BO_3_ units with exceptional rotation and deformation flexibility. By combining iDPC‐STEM, O K‐edge XAS, FTIR, ^11^B solid‐state NMR techniques, and the associated DFT calculations, we provide direct evidence that oxygen vacancies are stabilized through the formation of B_2_O_5_ units rather than BO_2_ species. Variable‐temperature neutron PDF analysis coupled with RMC modeling, together with AIMD simulations, further reveals that long‐range oxide‐ion migration is mediated by dynamic oxygen exchange between minimal BO_3_ and B_2_O_5_ units, with transient BO_2_ configurations as intermediates. This minimal and flexible transport network minimizes the steric constraints and decouples ion migration from large‐amplitude lattice relaxations, enabling efficient oxide‐ion transport with a high oxide‐ion conductivity of ∼ 1.5 × 10^−2^ S/cm at 1000°C, alongside excellent thermo‐mechanical stability across a wide range of oxygen partial pressures. Beyond advancing the understanding of atomic‐scale oxygen defect stabilization and migration, this work highlights minimal transport units combined with lightweight chemistry as a general strategy for developing next‐generation oxide‐ion electrolytes for portable and lightweight energy‐conversion technologies.

## Author Contributions

**Xiaohui Li**: conceptualization, data curation, formal analysis, investigation, supervision, writing – original draft, writing – review and editing, funding acquisition. **Yongkang Zheng**: software, data curation, formal analysis. **Li Yang**: formal analysis, data curation. **Xiaoge Wang**: formal analysis, data curation. **Yuan Gou**: data curation, formal analysis. **Guanqun Cai**: formal analysis, data curation. **Jie Liu**: formal analysis, data curation. **Cheng Li**: formal analysis, data curation. **Kun Lin**: formal analysis, data curation. **Qiang Li**: formal analysis, data curation. **Mingxue Tang**: formal analysis, data curation. **Junliang Sun**: formal analysis, data curation. **Xianran Xing**: formal analysis, data curation.**Xiaojun Kuang**: data curation, methodology, conceptualization, investigation, formal analysis, supervision, funding acquisition, writing – review and editing, writing – original draft.

## Conflicts of Interest

The authors declare no conflicts of interest.

## Supporting information




**Supporting File 1**: advs77283‐sup‐0001‐SuppMat.docx.

**Supporting File 2**: advs77283‐sup‐0002‐Video1.mp4.

## Data Availability

The data that support the findings of this study are available from the corresponding author upon reasonable request.
