# Supplementary material for: Minimal Transport Units Govern Oxygen‐Defect Stabilization and Transport for Lightweight Solid Electrolytes
Source: Adv Sci (Weinh). 2026 Sep 6:e77283. Online ahead of print. doi: 10.1002/advs.77283 (PMC13547790; doi:10.1002/advs.77283)
Supplement: Supplementary file 1 [file ADVS-9999-e77283-s002.docx]

**Supporting Information for:**

**Minimal Transport Units Govern Oxygen-Defect Stabilization and Transport for Lightweight Solid Electrolytes**

Xiaohui Li,^a*^ Yongkang Zheng,^a^ Li Yang,^b^ Xiaoge Wang,^c^ Yuan Gou,^a^ Guanqun Cai,^c^ Jie Liu,^d^ Cheng Li,^e^ Kun Lin,^f^ Qiang Li,^f^ Mingxue Tang,^d,g^ Junliang Sun,^c^ Xianran Xing.^f^ Xiaojun Kuang.^a,h^*

^a^ Guangxi Key Laboratory of Electrochemical and Magnetochemical Functional Materials, College of Chemistry and Bioengineering, Guilin University of Technology, Guilin 541004, People’s Republic of China.

^b^ MOE Key Laboratory of New Processing Technology for Nonferrous Metal and Materials, Guangxi Key Laboratory of Optical and Electronic Materials and Devices, College of Materials Science and Engineering, Guilin University of Technology, Guilin 541004, People’s Republic of China.

^c^ College of Chemistry and Molecular Engineering, Peking University, Beijing National Laboratory for Molecular Science (BNLMS), Beijing 100871, People’s Republic of China.

^d^ Center for High Pressure Science and Technology Advanced Research, Beijing, 100193, People's Republic of China.

^e^ Oak Ridge National Laboratory, Neutron Sciences Directorate, Oak Ridge, TN, 37831-2008, United States of America.

^f^ Beijing Advanced Innovation Center for Materials Genome Engineering, Institute of Solid State Chemistry, University of Science and Technology Beijing, Beijing 100083, People’s Republic of China.

^g^ School of Materials Science and Engineering, University of Science and Technology Beijing, Beijing 100083, People’s Republic of China.

^h^ Future Energy Interdisciplinary Center, School of Intelligent Manufacturing and Future Energy, Gannan Normal University, Ganzhou, Jiangxi 341000, People’s Republic of China.

E-mail address: xiaohuili@glut.edu.cn; [kuangxj@glut.edu.cn](mailto:Kuangxj@glut.edu.cn)

## Materials and Methods

**Experimental section.**

Polycrystalline samples of Sc_1-_*_x_*Zn*_x_*BO_3-_*_x_*_/2_ (0 ≤ *x* ≤ 0.35) were synthesized by the high temperature solid state reaction method. Sc_2_O_3_ (99.99%, Aladdin), ZnO (99.99%, Aladdin), and H_3_BO_3_ (99.99%, Aladdin) were used as starting materials, which were weighed according to the stoichiometric ratios, with H_3_BO_3_ in excess by 60% mol to compensate for volatilization at high temperatures. The materials were mixed in ethanol using a mortar and pestle. The resulting powders were pre-fired at 500 ºC for 6 h, and reground and pressed into pellets, and then sintered at 1100 ºC for 8 h to obtain the final ceramics with relative densities of ~73.7%. Moreover, owing to the large absorption of the naturally abundant ^10^B isotope to the neutrons, ^11^B isotope-rich raw material of H_3_^11^BO_3_ (99.99%, Aladdin) was used to synthesize Sc_0.7_Zn_0.3_^11^BO_2.85_ samples for the collection of neutron powder diffraction (NPD) data and neutron pair distribution function (nPDF) data.

**Characterization Methods**

Phase purity was checked by powder X-ray diffraction (XRD) using a PANAlytical X'Pert PRO diffractometer with Cu Kα radiation operating at 45 kV and 40 mA. Variable temperature XRD patterns were collected on this instrument equipped with an Anton Parr HTK 1200N high temperature attachment. The time-of-flight (TOF) NPD data and total scattering nPDF data (*Q*_max_ = 29.9955 Å^-1^) at room temperature were performed on the POWGEN and BL-1B NOMAD beamlines at Oak Ridge National Laboratory of Spallation Neutron Source (SNS), respectively. Variable temperature total scattering nPDF data (*Q*_max_ = 25 Å^-1^) were collected on a Multi-Physics Instrument (MPI) at the China Spallation Neutron Source.[1] The average lattice fitting was conducted based on PDFgui software.[2] Reverse Monte Carlo simulations for the nPDF data were carried out by RMCprofile software[3] combining the atomic movements and swapping, which utilizes a 16 × 16 × 5 (76.12Å × 76.12 Å × 76.61 Å) large supercell containing 38400 atoms (including 5514 Sc atoms, 2166 Zn atoms, 7680 B atoms, 18847 O atoms, 4193 O vacancies). TOPAS[4] and Jana 2006 software[5] were used to perform Rietveld analysis of XRD and NPD data. The rotation electron diffraction (RED) data was collected on a JEOL-2100 electron microscope operating at 200 kV using a single-tilt tomography sample holder, and REDp processing software[6] was used to process the RED data.

The morphology images and X-ray energy-dispersive spectroscopy (EDS) data were collected on a FEI Tecnai-G2 field emission transmission electron microscope (TEM) equipped with a Li/Si EDX detector and on a Hitachi (Tokyo, Japan) S4800 scanning electron microscope (SEM). The ultrasoft X-ray absorption spectra (XAS) were collected on 4B9B beamline of the photoelectron spectroscopy station at Beijing Synchrotron Radiation Facility (BSRF). Fourier-Transform Infrared (IR) spectra were recorded on a Tensor 27 spectrometer using the OPUS/IR system within the region of 4000-400 cm^-1^. The composition of Sc_0.7_Zn_0.3_BO_3_ was performed on the inductively coupled plasma-atomic emission spectrometry (ICP-OES, iCAP 7600, Thermo Fisher, Germany).

Alternating current (AC) impedance spectroscopy measurements were performed on a Solartron 1260 frequency response analyzer over a frequency range from 10^-1^ to 10^7^ Hz. Pt paste was coated on the top and bottom surfaces of the ceramics and fired at 750 ºC for 0.5 h to remove the organic compositions to form Pt electrodes. The oxygen transport numbers ($t_{O^{2-}}$) were acquired by oxygen concentration cell method through measuring the electromotive force (EMF) under different oxygen partial pressures (1% O_2_ // pure O_2_) over a temperature range of 500-1000 ºC.

Solid-state ^11^B nuclear magnetic resonance (NMR) spectra were collected on Bruker advance III spectrometer equipped with WVT 4 mm CP/MAS double response probe head operating at magnetic fields of 9.4 T. ssNake software[7] was used to perform the deconvolution of the experimental solid-state ^11^B NMR spectra, which allows for inclusion of the quadrupolar interaction.

**DFT-based ^11^B NMR calculations.** Periodic DFT calculations for geometry optimization and the evaluation of chemical shielding and electric field gradient (EFG) tensors were performed using the CASTEP code[8] implemented in Materials Studio 2020. The structural optimization of atomic positions and cell parameters was performed using ultra-soft pseudopotentials (USPPs)[9], where the chosen mechanical criterion is 3.0×10^-2^ eV·Å^-1^ and the energy criterion is 10^-5^ eV. The plane-wave basis cut-off energy is set as 340 eV and the Brillouin zone is set as a Monkhorst-Packed 1×1×1 grid used for sampling. The electric field gradient and the NMR chemical shielding tensor were calculated using the projector augmented wave (PAW)[10] and gauge included projector augmented wave (GIPAW) methods[11], respectively. According to the previous studies on the ^11^B solid-state NMR parameters,[12] the ^11^B isotropic chemical shift (*δ*_iso_) can be derived from the calculated isotropic shielding values (σ_iso_), according to the formula of *δ*_iso_ = -1.0071×σ_iso_ + 94.62. For structural modeling, a 2 × 2 × 1 supercell of Sc_24_B_24_O_72_ was constructed based on the initial structure of ScBO_3_ (Space group: *R*$\bar{3}$*c*). Zn^2+^ ions were substituted onto Sc^3+^ sites, and the corresponding number of oxygen atoms was removed to maintain charge neutrality. Specifically, the Zn_2_Sc_22_B_24_O_71_, Zn_4_Sc_20_B_24_O_70_, and Zn_6_Sc_18_B_24_O_69_ models contain two, four, and six Zn substitutions and one, two, and three oxygen vacancies, respectively. For each composition, oxygen vacancies were introduced at crystallographically reasonable oxygen sites, and different chemically plausible and symmetry-distinct Zn/vacancy arrangements were considered within the finite supercell. In total, 6, 4, and 3 representative configurations were constructed for Zn_2_Sc_22_B_24_O_71_, Zn_4_Sc_20_B_24_O_70_ and Zn_6_Sc_18_B_24_O_69_, respectively. These configurations were selected to sample, as comprehensively as practicable, the local configurational diversity associated with aliovalent Zn substitution and oxygen-vacancy formation. All models were fully relaxed, and the resulting B–O coordination environments were determined after geometry optimization rather than imposed in the initial structures.

**^11^B solid state NMR spectrum simulation.** ^11^B NMR spectra were simulated using WSolids software package[13] using the calculated chemical shifts (*δ*), quadrupolar coupling parameters (*C*_Q_) and asymmetry parameters (*η*) for each B site. Mixed Gaussian (200 Hz) and Lorentzian (300 Hz) line types with a mixing ratio of 1:1 was used for the simulated lines. Each calculated B site is treated as an independent NMR signal for modeling the simulated ^11^B NMR spectrum, and the peak of each calculated B site is fixed to the same intensity and width using a mixed Gaussian and Lorentz line shape. The simulated ^11^B NMR spectrum for each composition/structural model is the sum of all calculated ^11^B peaks, in which each ^11^B peak contributes equally.

**Ab initio molecular dynamics (AIMD) simulations.** AIMD simulations were performed to investigate oxide-ion migration in Sc_1-_*_x_*Zn*_x_*BO_3-_*_x_*_/2_ using projected augmented wave (PAW) pseudopotentials[10] included in the Vienna *ab initio* simulation package (VASP).[14] Perdew-Berke-Erzerhof (PBE)[15] functional within the generalized gradient approximation (GGA) was adopted for the exchange correlation function. The cutoff energies were set as 520 eV and 400 eV for the geometric optimization and *ab initio* MD simulations, respectively. The sampling of the Brillouin zone in geometric optimizations were performed using a 9×9×9 and 4×3×2 *k*-point meshes for the primitive cell and the supercell for *ab initio* MD simulations, respectively. A 2 × 2 × 1 supercell containing 117 atoms (18 Sc, 6 Zn, 24 B atoms, and 69 O atoms) was constructed based on the ScBO_3_ framework (Space group: *R*$\bar{3}$*c*). The resulting supercell corresponds to the composition Sc_0.75_Zn_0.25_BO_2.875_. The oxygen-vacancy defect model was generated by substituting two Sc atoms with Zn atoms and removing one oxygen atom from a neighboring BO_3_ unit to maintain charge compensation. Among the generated defect configurations, the lowest-energy configuration containing B_2_O_5_ unit was selected as the starting structure for AIMD simulations. The AIMD simulations were performed in the NVT ensemble using a Nosé thermostat at 1473, 1573, and 1673 K. A time step of 2 fs was employed, and each AIMD trajectory was propagated for 40,000 steps, corresponding to a total simulation time of 80 ps at each temperature. The comparation of experimental and calculated structural parameters are given in Table S1. For the high temperature *ab initio* MD simulations, the Brillouin zone was sampled with a 1×1×1 *k*-point mesh at the gamma point. The MD data analysis was carried out using the Visual Molecular Dynamics packages,[16] and the mean square displacement data were exported using VASPKIT package.[17]

**Table S1.** Comparison of experimental (exp.) and calculated (calc.) cell parameters, bond lengths and bond angles for ScBO_3_.

| ScBO_3_ (Space group: *R*$\bar{\boldsymbol{3}}$*c*) | | | |
| --- | --- | --- | --- |
| Parameters | Calc. | Exp. | Δ(Calc.-Exp.) |
| *a* = *b* (Å) | 4.8047 | 4.7480 | 0.0567 |
| *c* (Å) | 15.436 | 15.262 | 0.174 |
| *α* = *β* | 90 | 90 | 0 |
| *γ* | 120 | 120 | 0 |
| Volume (Å^3^) | 308.60 | 297.96 | 10.64 |
| Sc-O1 (×6) (Å) | 2.1462 | 2.1199 | 0.0263 |
| B1-O1 (×3) (Å) | 1.3885 | 1.3751 | 0.0134 |
| O1-Sc-O1 (×6) (º) | 87.777 | 87.713 | 0.064 |
| O1-Sc-O1 (×6) (º) | 92.223 | 92.287 | -0.064 |
| O1-Sc-O1 (×3) (º) | 180.00 | 180.00 | 0 |
| O1-B1-O1(×3) (º) | 120.00 | 120.00 | 0 |

**Table S2.** The final refined structural parameters of Sc_0.7_Zn_0.3_BO_2.85_ from XRD data*

| Atom | Wyckoff site | *x* | *y* | *z* | *occ.* | B_iso_ (Å^2^) |
| --- | --- | --- | --- | --- | --- | --- |
| Sc1 | 6*b* | 0 | 0 | 0 | 0.738 (3) | 1 |
| Zn1 | 6*b* | 0 | 0 | 0 | 0.262 (3) | 1 |
| B1 | 6*a* | 0 | 0 | 0.25 | 1 | 1 |
| O1 | 18*e* | 0.7104(3) | 0 | 0.25 | 0.96(5) | 1 |

*Space group: *R*-$\bar{3}$*C*, *a* = *b* = 4.75429 (7) Å, *c* = 15.2886 (4) Å, *α* = *β* = 90 º, *γ* = 120 º, *V* = 299.275 (1) Å^3^, *Z* = 6.

**Figure S1.** (a) TEM mapping and (b) EDS spectrum of parent ScBO_3_.

**Figure S2.** TEM-EDS spectrum of Sc_0.85_Zn_0.15_BO_2.925_.

**Figure S3.** TEM-EDS spectrum of Sc_0.7_Zn_0.3_BO_2.85_.

**Figure S4.** SEM image (a) and Mapping (b-f) of Sc_0.7_Zn_0.3_BO_2.85_.

**Figure S5.** Typical complex impedance plot of parent ScBO_3_ at 900 ºC. The data was fitted (red line) using an equivalent circuit comprising the contributions of the bulk and ground boundary responses, both of which are parallel *RC* elements, where *R*_b_ and *C*_b_ (~10^-12^ F/cm) are bulk resistance and capacitance, respectively, and *R*_gb_ and *C*_gb_ (~10^-11^ F/cm) are grain boundary and capacitance.

**Supplementary Note 1.** Basis and physical interpretation of the equivalent-circuit analysis.

The equivalent-circuit model was established on the basis of the characteristic features of the Nyquist plots and the impedance behavior generally observed in polycrystalline ceramic electrolytes. In real ceramic systems, ideal Debye-type relaxation is rarely achieved because of microstructural heterogeneity, grain-boundary effects, and a distribution of relaxation times. As a result, the impedance arcs are often depressed rather than perfectly semicircular. Constant-phase elements (CPEs) were therefore introduced to represent these non-ideal capacitive responses, consistent with established descriptions of impedance relaxation and universal dielectric behavior.[18] Within the adopted circuit, the high-frequency and intermediate-frequency responses are assigned to the bulk and grain-boundary contributions, respectively. These assignments are supported by the corresponding capacitance values, which fall within the characteristic ranges generally associated with intrinsic lattice and grain-boundary processes. The low-frequency Warburg-type contribution is attributed to processes occurring at the electrode/electrolyte interface, where ion accumulation and diffusion become dominant under ion-blocking electrode conditions.[19] Accordingly, the selected equivalent circuit enables the bulk, grain-boundary, and electrode/electrolyte interfacial contributions to be distinguished in a physically meaningful manner. Comparable equivalent-circuit models have been extensively applied to ceramic ionic conductors and related electroceramic systems.[20-22]

**Figure S6.** Oxygen transport numbers of parent ScBO_3_.

**Figure S7.** The variations of cell parameters as a function of temperature for Sc_0.7_Zn_0.3_BO_2.85_.

**Table S3.** Anisotropic atomic displacement parameters of Sc_0.7_Zn_0.3_BO_2.85_ from NPD data at 298 K.

| Atom | Site | U_11_(Å^2^) | U_22_(Å^2^) | U_33_(Å^2^) | U_12_(Å^2^) | U_13_(Å^2^) | U_23_(Å^2^) |
| --- | --- | --- | --- | --- | --- | --- | --- |
| Sc1 | 6*b* | 0.00222(12) | 0.00222(12) | 0.00342(14) | 0.00111(6) | 0 | 0 |
| Zn1 | 6*b* | 0.00222(12) | 0.00222(12) | 0.00342(14) | 0.00111(6) | 0 | 0 |
| B1 | 6*a* | 0.00188(18) | 0.00188(18) | 0.0043(3) | 0.00094(9) | 0 | 0 |
| O1 | 18*e* | 0.00210(13) | 0.00409(15) | 0.00595(15) | 0.00205(8) | -0.00115(6) | -0.0023(1) |

**Table S4.** The final refined structural parameters of Sc_0.7_Zn_0.3_BO_2.85_ from NPD data at 873 K.

| Atom | Wyckoff site | *x* | *y* | *z* | occ. | U_iso_ (Å^2^) |
| --- | --- | --- | --- | --- | --- | --- |
| Sc1 | 6*b* | 0 | 0 | 0 | 0.727(6) | 0.0117 |
| Zn1 | 6*b* | 0 | 0 | 0 | 0.273 (6) | 0.0194 |
| B1 | 6*a* | 0 | 0 | 0.25 | 1 | 0.0046 |
| O1 | 18*e* | 0.28858(18) | 0 | 0.25 | 0.923(7) | 0.0104 |

Space group: *R*-$\bar{3}$*C*, *a* = *b* = 4.7724(2) Å, *c* = 15.4355 (5) Å, *α* = *β* = 90 º*, γ* = 120 º, *V* = 304.45 (1) Å^3^.

**Table S5.** Anisotropic atomic displacement parameters of Sc_0.7_Zn_0.3_BO_2.85_ from NPD data at 873 K.

| Atom | Site | U_11_(Å^2^) | U_22_(Å^2^) | U_33_(Å^2^) | U_12_(Å^2^) | U_13_(Å^2^) | U_23_(Å^2^) |
| --- | --- | --- | --- | --- | --- | --- | --- |
| Sc1 | 6*b* | 0.011(6) | 0.011(6) | 0.013(3) | 0.0056(30) | 0 | 0 |
| Zn1 | 6*b* | 0.02 (4) | 0.02 (4) | 0.028(25) | 0.008(20) | 0 | 0 |
| B1 | 6*a* | 0.0030(9) | 0.0030(9) | 0.0079(8) | 0.0015(5) | 0 | 0 |
| O1 | 18*e* | 0.0081(8) | 0.0121(7) | 0.0124(6) | 0.0061(3) | -0.0037(19) | -0.007(4) |

**Figure S8.** The XAS spectra of Au grid as a reference for collecting XAS spectra of each sample.

**Figure S9.** Electronic calculations of parent ScBO_3_ and Sc_0.7_Zn_0.3_BO_2.85_. (a) Total density of states of O-2p for parent ScBO_3_ and Sc_0.7_Zn_0.3_BO_2.85_. (b) Partial density of states of O-2p for Sc_0.7_Zn_0.3_BO_2.85_. Band structures of (c) parent ScBO_3_ and (d) Sc_0.7_Zn_0.3_BO_2.85_.

**Table S6.** Chemical shift *δ* and quadrupole coupling parameter *C*_Q_ values of resonance signals from the deconvolution of experimental ^11^B NMR spectra of H_3_BO_3_ and Zn*_x_*Sc_1-_*_x_*BO_3-0.5_*_x_* (*x* = 0, 0.1, 0.2, 0.3).*

| Samples | Parameters | R1 | R2 |
| --- | --- | --- | --- |
| H_3_BO_3_ | *δ* (ppm)  *C*_Q_ (MHz)  Integral (%) | 19.60  2.56  100 | /  /  / |
| ScBO_3_ | *δ* (ppm)  *C*_Q_ (MHz)  Integral (%) | 21.29  2.77  100 | /  /  / |
| Zn_0.1_Sc_0.9_BO_2.95_ | *δ* (ppm)  *C*_Q_ (MHz)  Integral (%) | 21.29  2.78  91.81 | 0.50  0.13  8.19 |
| Zn_0.2_Sc_0.8_BO_2.9_ | *δ* (ppm)  *C*_Q_ (MHz)  Integral (%) | 20.4  2.70  79.84 | 0.50  0.13  20.16 |
| Zn_0.3_Sc_0.7_BO_2.85_ | *δ* (ppm)  *C*_Q_ (MHz)  Integral (%) | 20.13  2.69  71 | 0.52  0.13  29 |

*: “/” means not applicable. Integral denotes the proportion of the integrated intensity for each resonance signal of the B nucleus.

**Table S7.** Total and relative energies and B coordination environments for different structural models after geometric optimization for Sc_1-_*_x_*Zn*_x_*BO_3-_*_x_*_/2_.

| Composition | Structural model number | Total Energy  /eV | Final Energy  /eV | Coordination environments |
| --- | --- | --- | --- | --- |
| Sc_24_B_24_O_72_ | 1 | -1479570 | -64160.22 | BO_3_ |
| Zn_2_Sc_22_B_24_O_71_ | 1 | -1489180 | -64576.86 | BO_3_, BO_2_ |
|  | 2 | -1489170 | -64576.63 | BO_3_, BO_2_ |
|  | 3 | -1489160 | -64576.26 | BO_3_, BO_2_ |
|  | 4 | -1489160 | -64576.20 | BO_3_, BO_2_ |
|  | 5 | -1489170 | -64576.49 | BO_3_, BO_2_ |
|  | 6 | -1489170 | -64576.34 | BO_3_, BO_2_ |
| Zn_4_Sc_20_B_24_O_70_ | 1 | -1498700 | -64989.66 | BO_3_, BO_2_, B_2_O_5_ |
|  | 2 | -1498670 | -64989.53 | BO_3_, BO_2_ |
|  | 3 | -1498670 | -64988.33 | BO_3_, BO_2_ |
|  | 4 | -1498700 | -64989.76 | BO_3_, BO_2_, B_2_O_5_ |
| Zn_6_Sc_18_B_24_O_69_ | 1 | -1508290 | -65404.61 | BO_3_, BO_2_, B_2_O_5_ |
|  | 2 | -1508250 | -65403.72 | BO_3_, BO_2_ |
|  | 3 | -1508270 | 65404.57 | BO_3_, BO_2_ |

**Table S8.** The calculated ^11^B chemical shifts of BO_2/3/4_ units in Sc_1-_*_x_*Zn*_x_*BO_3-_*_x_*_/2_. ^*^

| Composition | $B^{CE}$ | | $\delta^{MIN}$/ppm | $\delta^{MAX}$/ppm | $\delta^{AV}$/ppm | Number |
| --- | --- | --- | --- | --- | --- | --- |
| Sc_24_B_24_O_72_ | BO_3_ | | 21.10 | 21.11 | 21.11 | 24 |
| Zn_2_Sc_22_B_24_O_71_ | BO_2_ | | 8.75 | 9.74 | 9.02 | 5 |
|  | BO_3_, Zn | | 20.14 | 21.65 | 20.93 | 55 |
|  | BO_3_ | | 20.46 | 21.61 | 21.22 | 60 |
| Zn_4_Sc_20_B_24_O_70_ | BO_2_ | 9.37 | | 10.64 | 10.14 | 6 |
|  | BO_3_, Zn | 18.92 | | 21.92 | 21.02 | 59 |
|  | BO_3_ | 20.59 | | 22.06 | 21.36 | 27 |
|  | B_2_O_5_ | 18.06 | | 20.37 | 19.22 | 4 |
| Zn_6_Sc_18_B_24_O_69_ | BO_2_ | | 9.52 | 11.12 | 10.34 | 8 |
|  | BO_3_, Zn | | 19.32 | 22.59 | 21.09 | 58 |
|  | BO_3_ | | 21.91 | 22.42 | 22.18 | 4 |
|  | B_2_O_5_ | | 17.15 | 21.34 | 19.25 | 2 |

^*^ CE, AV, MIN, MAX, and number represent the coordination environment, average value, minimum value, maximum value, and number of ^11^B sites in BO_2/3/4_ units with different second coordination environments, respectively.

**Table S9.** Average calculated ^11^B chemical shifts (δ), quadrupolar coupling parameters (*C*_Q_), and asymmetry parameters ($\eta_{Q}$) in Sc_1-_*_x_*Zn*_x_*BO_3-_*_x_*_/2_.

| $B^{CE}$ | δ/ppm | C_Q_/MHz | $\eta_{Q}$ | number |
| --- | --- | --- | --- | --- |
| BO_2_ | 9.93 | -4.3$\pm0.1$ | 0.01$\pm0.01$ | 19 |
| BO_3_,Zn | 21.01 | 3.1$\pm0.1$ | 0.15$\pm0.1$ | 172 |
| BO_3_ | 21.26 | 3.1$\pm0.1$ | 0.2$\pm0.1$ | 115 |
| B_2_O_5_ | 19.23 | 3.0$\pm0.1$ | 0.5$\pm0.1$ | 6 |

**Figure S10.** Simulated ^11^B NMR spectra of (a) parent ScBO_3_ and (b-c) Sc_1-_*_x_*Zn*_x_*BO_3-_*_x_*_/2_ (*x* = 0.083, 0.167) compositions with contributions from different structural units containing BO_2_ units, where (BO_3_, Zn) units denote BO_3_ units with different numbers of Zn atom neighbors in the second coordination spheres.

**Table S10.** Calculated ^11^B chemical shifts (δ), quadrupolar coupling parameters (*C*_Q_), and asymmetry parameters ($\eta_{Q}$) for Sc_1-_*_x_*Zn*_x_*BO_3-_*_x_*_/2_ with *x* = 0.083 (Zn_2_Sc_22_B_24_O_71_), based on the lowest-energy structural model.

| Nucleus | Chemical Shift (ppm) | *C*_Q_ (MHz) | $\eta_{Q}$ |
| --- | --- | --- | --- |
| B1 | 21.19234 | 3.057 | 0.13 |
| B2 | 21.35348 | 3.101 | 0.01 |
| B3 | 21.40383 | 3.095 | 0.03 |
| B4 | 20.53772 | 3.079 | 0.22 |
| B5 | 20.90028 | 3.104 | 0.03 |
| B6 | 20.87007 | 3.084 | 0.22 |
| B7 | 21.08156 | 3.117 | 0.04 |
| B8 | 21.51461 | 3.107 | 0.03 |
| B9 | 21.54482 | 3.115 | 0.1 |
| B10 | 21.27291 | 3.098 | 0.06 |
| B11 | 21.28298 | 3.081 | 0.11 |
| B12 | 21.20241 | 3.076 | 0.09 |
| B13 | 20.90028 | 3.094 | 0.11 |
| B14 | 20.42694 | 3.11 | 0.09 |
| B15 | 21.4139 | 3.086 | 0.05 |
| B16 | 20.44709 | 3.088 | 0.1 |
| B17 | 21.52468 | 3.098 | 0.02 |
| B18 | 21.33333 | 3.106 | 0.04 |
| B19 | 8.925861 | -4.402 | 0.02 |
| B20 | 20.79957 | 3.105 | 0.14 |
| B21 | 21.28298 | 3.095 | 0.13 |
| B22 | 21.26284 | 3.096 | 0.05 |
| B23 | 21.1722 | 3.093 | 0.03 |
| B24 | 20.83985 | 3.084 | 0.1 |

**Table S11.** Calculated ^11^B chemical shifts (δ), quadrupolar coupling parameters (*C*_Q_), and asymmetry parameters ($\eta_{Q}$) for Sc_1-_*_x_*Zn*_x_*BO_3-_*_x_*_/2_ with *x* = 0.167 (Zn_4_Sc_20_B_24_O_70_), based on the lowest-energy structural model.

| Nucleus | Chemical Shift (ppm) | *C*_Q_ (MHz) | $\eta_{Q}$ |
| --- | --- | --- | --- |
| B1 | 20.93049 | 3.079 | 0.07 |
| B2 | 21.07149 | 3.081 | 0.12 |
| B3 | 20.89021 | 3.051 | 0.29 |
| B4 | 18.06026 | 2.913 | 0.53 |
| B5 | 20.39673 | 3.081 | 0.17 |
| B6 | 21.04127 | 3.111 | 0.17 |
| B7 | 20.82978 | 3.097 | 0.14 |
| B8 | 21.43404 | 3.085 | 0 |
| B9 | 21.59518 | 3.098 | 0.19 |
| B10 | 21.06142 | 3.072 | 0.22 |
| B11 | 20.31616 | 3.075 | 0.51 |
| B12 | 21.68582 | 3.063 | 0.1 |
| B13 | 21.31319 | 3.096 | 0.11 |
| B14 | 20.44709 | 3.098 | 0.12 |
| B15 | 21.1017 | 3.086 | 0.05 |
| B16 | 18.91629 | 3.04 | 0.21 |
| B17 | 21.94766 | 3.088 | 0.02 |
| B18 | 21.47433 | 3.089 | 0.02 |
| B19 | 9.368985 | -4.4 | 0.03 |
| B20 | 20.89021 | 3.096 | 0.15 |
| B21 | 20.84993 | 3.064 | 0.32 |
| B22 | 21.00099 | 3.077 | 0.17 |
| B23 | 21.15206 | 3.061 | 0.17 |
| B24 | 21.31319 | 3.079 | 0.17 |

**Table S12.** Calculated ^11^B chemical shifts (δ), quadrupolar coupling parameters (*C*_Q_), and asymmetry parameters ($\eta_{Q}$) for Sc_1-_*_x_*Zn*_x_*BO_3-_*_x_*_/2_ with *x* = 0.25 (Zn_6_Sc_18_B_24_O_69_), based on the lowest-energy structural model.

| Nucleus | Chemical Shift (ppm) | *C*_Q_ (MHz) | $\eta_{Q}$ |
| --- | --- | --- | --- |
| B1 | 21.94766 | 3.028 | 0.1 |
| B2 | 21.24269 | 3.047 | 0.08 |
| B3 | 20.73914 | 3.091 | 0.05 |
| B4 | 17.15387 | 3.007 | 0.41 |
| B5 | 20.55787 | 3.055 | 0.25 |
| B6 | 11.12134 | -4.361 | 0.02 |
| B7 | 21.3434 | 3.151 | 0.49 |
| B8 | 21.57504 | 3.069 | 0.15 |
| B9 | 19.31913 | 3.063 | 0.15 |
| B10 | 21.60525 | 3.114 | 0.04 |
| B11 | 20.75929 | 3.115 | 0.04 |
| B12 | 21.27291 | 3.067 | 0.11 |
| B13 | 20.82978 | 3.08 | 0.04 |
| B14 | 20.69886 | 3.087 | 0.04 |
| B15 | 21.63546 | 3.039 | 0.19 |
| B16 | 22.20951 | 3.09 | 0.09 |
| B17 | 21.90738 | 3.102 | 0.06 |
| B18 | 21.46426 | 3.071 | 0.19 |
| B19 | 10.62786 | -4.354 | 0.01 |
| B20 | 20.4773 | 3.07 | 0.21 |
| B21 | 20.98085 | 3.082 | 0.13 |
| B22 | 21.50454 | 3.071 | 0.14 |
| B23 | 21.45419 | 3.088 | 0.14 |
| B24 | 21.18227 | 3.058 | 0.17 |

**Figure S11.** Simulated ^11^B NMR spectra of (a) parent ScBO_3_ and (b-d) Sc_1-_*_x_*Zn*_x_*BO_3-_*_x_*_/2_ with *x* = 0.083, 0.167, and 0.25, respectively, based on the lowest-energy structural model for each composition. Contributions from all B coordination environments are included, and the BO_2_-related resonances are indicated by blue arrows.

**Figure S12.** AIMD simulations of Sc_0.7_Zn_0.3_BO_2.85_ at 1473 K. (a) Mean square displacements of atoms. (b-d) Scatter plot of atoms: (b) Oxygens and cations; (c) oxygens; (d) cations. (e-h) Temporal evolution of B-O configurations.

**Figure S13.** AIMD simulations of Sc_0.7_Zn_0.3_BO_2.85_ at 1573 K. (a) Mean square displacements of atoms. (b-d) Scatter plot of atoms: (b) Oxygens and cations; (c) oxygens; (d) cations. (e-h) Temporal evolution of B-O configurations.

## References

[1]	J. P. Xu, Y. G. Xia, Z. D. d. Li, H. C. Chen, X. L. Wang, Z. Z. Sun, W. Yin, Multi-physics instrument: Total scattering neutron time-of-flight diffractometer at China Spallation Neutron Source, *Nuclear Inst. and Methods in Physics Research, A* **2021**, *1013*, 165642.

[2]	C. L. Farrow, P. Juhas, J. W. Liu, D. Bryndin, E. S. Božin, J. Bloch, T. Proffen, S. J. L. Billinge, PDFfit2 and PDFgui: computer programs for studying nanostructure in crystals, *J. Phys.: Condens. Matter* **2007**, *19* (33), 335219.

[3]	M. G. Tucker, D. A. Keen, M. T. Dove, A. L. Goodwin, Q. Hui, RMCProfile: reverse Monte Carlo for polycrystalline materials, *J. Phys.: Condens. Matter* **2007**, *19* (33), 335218.

[4]	A. Bruker, *TOPAS V3: General profile and structure analysis software for powder diffraction data*, Bruker AXS, Karlsruhe Germany **2005**.

[5]	Michal. Dušek, Václav. Petříček, Lukáš. Palatinus, Jan. Rohlíček, Karla. Fejfarová, Václav. Eigner, Monika. Kučeráková, Morgane. Poupon, Margarida. Henriques, Jakub. Plášil, Eliška. Skořepová, *Jana 2006 Cookbook*, Czech Republic, Institute of Physics ASCR, v.v.i., Na Slovance 2, Prague, **2019**.

[6]	W. Wan, J. Sun, J. Su, S. Hovmoller, X. Zou, Three-dimensional rotation electron diffraction: software RED for automated data collection and data processing, *J. Appl. Crystallogr.* **2013**, *46* (Pt 6), 1863.

[7]	S. G. J. van Meerten, W. M. J. Franssen, A. P. M. Kentgens, ssNake: A cross-platform open-source NMR data processing and fitting application, *J. Magn. Reson.* **2019**, *301*, 56.

[8]	S. J. Clark, M. D. Segall, C. J. Pickard, P. J. Hasnip, M. I. J. Probert, K. Refson, M. C. Payne, First Principles Methods Using CASTEP., *Z. Kristallogr.* **2005**, *220*, 567−570.

[9]	J. R. Yates, C. J. Pickard, F. Mauri, Calculation of NMR chemical shifts for extended systems using ultrasoft pseudopotentials, *Phys. Rev. B.* **2007**, *76* (2), 024401.

[10]	P. E. Blochl, Projector augmented-wave method, *Phys. Rev. B.* **1994**, *50* (24), 17953.

[11]	C. J. Pickard, F. Mauri, All-electron magnetic response with pseudopotentials: NMR chemical shifts, *Phys. Rev. B.* **2001**, *63* (24), 245101.

[12]	J. Czernek, J. Brus, On the predictions of the ^11^B solid state NMR parameters, *Chem. Phys. Lett.* **2016**, *655-656*, 66.

[13]	K. Eichele, R. E. Wasylishen, *WSolids: Solid-State NMR Spectrum Simulation*, Universitat tubingen, **2015.**

[14]	G. Kresse, J. Furthmuler, Efficient iterative schemes for ab initio total-energy calculations using a plane-wave basis set., *Physic. Rev. B.* **1996**, *54*, 11169.

[15]	J. P. Perdew, K. Burke, M. Ernzerhof, Generalized Gradient Approximation Made Simple, *Phys. Rev. Lett.* **1996**, *77*, 3865.

[16]	W. Humphrey, A. Dalke, K. Schulten, VMD: Visual Molecular Dynamics., *J. Mol. Graphics.* **1996**, *14*, 33.

[17]	V. Wang, N. Xu, J. C. Liu, G. Tang, W. T. Geng, A User-Friendly Interface Facilitating High-throughput Computing and Analysis Using VASP Code, *VASPKIT* **2019**.

[18]	J. S. Macdonald, Note on the parameterization of the constant-phase admittance element, *Solid State Ion.* **1984**, *13*, 147.

[19]	J. T. S. Irvine, D. C. Sinclair, A. R. West, Electroceramics : Characterization by Impedance Spectroscopy, *Adv. Mater.* **1990**, *2*, 132.

[20]	N. Masó, A. R. West, Electronic Conductivity in Yttria-Stabilized Zirconia under a Small dc Bias, *Chem. Mater.* **2015**, *27* (5), 1552.

[21]	J. Jamnik, J. Maier, Treatment of the Impedance of Mixed Conductors Equivalent Circuit Model and Explicit Approximate Solutions, *J. Electrochem. Soc.* **1999**, *146*, 4183.

[22]	N. Masó, X. Y. Yue, T. Goto, A. R. West, Frequency-dependent electrical properties of ferroelectric BaTi_2_O_5_ single crystal, *J. Appl. Phys.* **2011**, *109* (2), 024107.
